# Mechanisms of antigen escape from BCMA- or GPRC5D-targeted immunotherapies in multiple myeloma

**DOI:** 10.1038/s41591-023-02491-5

**Published:** 2023-08-31

**Authors:** Holly Lee, Sungwoo Ahn, Ranjan Maity, Noemie Leblay, Bachisio Ziccheddu, Marietta Truger, Monika Chojnacka, Anthony Cirrincione, Michael Durante, Remi Tilmont, Elie Barakat, Mansour Poorebrahim, Sarthak Sinha, John McIntyre, Angela M.Y. Chan, Holly Wilson, Shari Kyman, Amrita Krishnan, Ola Landgren, Wencke Walter, Manja Meggendorfer, Claudia Haferlach, Torsten Haferlach, Hermann Einsele, Martin K. Kortüm, Stefan Knop, Jean Baptiste Alberge, Andreas Rosenwald, Jonathan J. Keats, Leo Rasche, Francesco Maura, Paola Neri, Nizar J. Bahlis

**Affiliations:** 1 Arnie Charbonneau Cancer Institute, University of Calgary, Calgary, Alberta Canada; 2 Sylvester Comprehensive Cancer Center, Miami, FL USA; 3 MLL Munich Leukemia Laboratory, Munich, Germany; 4 Department of Comparative Biology and Experimental Medicine, Faculty of Veterinary Medicine, University of Calgary, Calgary, Alberta Canada; 5 Precision Oncology Hub Laboratory, Tom Baker Cancer Centre, Calgary, Alberta Canada; 6 Translational Genomics Research Institute, Phoenix, AZ USA; 7 City of Hope Comprehensive Cancer Center, Duarte, CA USA; 8 Department of Internal Medicine 2, University Hospital of Würzburg, Würzburg, Germany; 9 Department of Internal Medicine 5, Paracelsus Medical School, Nuremberg General Hospital, Nuremberg, Germany; 10 Harvard Medical School, Boston, MA USA; 11 Institute of Pathology, University of Würzburg, Würzburg, Germany; 12 Mildred Scheel Early Career Center, University Hospital of Würzburg, Würzburg, Germany

**Keywords:** Myeloma, Translational research

## Abstract

B cell maturation antigen (BCMA) target loss is considered to be a rare event that mediates multiple myeloma (MM) resistance to anti-BCMA chimeric antigen receptor T cell (CAR T) or bispecific T cell engager (TCE) therapies. Emerging data report that downregulation of G-protein-coupled receptor family C group 5 member D (GPRC5D) protein often occurs at relapse after anti-GPRC5D CAR T therapy. To examine the tumor-intrinsic factors that promote MM antigen escape, we performed combined bulk and single-cell whole-genome sequencing and copy number variation analysis of 30 patients treated with anti-BCMA and/or anti-GPRC5D CAR T/TCE therapy. In two cases, MM relapse post-TCE/CAR T therapy was driven by BCMA-negative clones harboring focal biallelic deletions at the *TNFRSF17* locus at relapse or by selective expansion of pre-existing subclones with biallelic *TNFRSF17* loss. In another five cases of relapse, newly detected, nontruncating, missense mutations or in-frame deletions in the extracellular domain of BCMA negated the efficacies of anti-BCMA TCE therapies, despite detectable surface BCMA protein expression. In the present study, we also report four cases of MM relapse with biallelic mutations of *GPRC5D* after anti-GPRC5D TCE therapy, including two cases with convergent evolution where multiple subclones lost *GPRC5D* through somatic events. Immunoselection of BCMA- or GPRC5D-negative or mutant clones is an important tumor-intrinsic driver of relapse post-targeted therapies. Mutational events on BCMA confer distinct sensitivities toward different anti-BCMA therapies, underscoring the importance of considering the tumor antigen landscape for optimal design and selection of targeted immunotherapies in MM.

## Abstract (web summary)

Tumor-intrinsic resistance mechanisms, including mutational events and immunoselection of mutant clones, were identified in 30 patients with multiple myeloma who experienced relapse after treatment with anti-BCMA CAR T cells and/or anti-BCMA bispecific T cell engagers (TCEs) or anti-GPRC5D TCE therapy.

## Main

Targeted immunotherapies, including CAR T and TCEs, enhance T cell-mediated elimination of tumors^1,2^. In MM, BCMA and GPRC5D are among key immunotherapeutic targets^3^. BCMA, encoded by the *TNFRSF17* gene on chromosome 16p, is a type III transmembrane domain protein of the tumor necrosis factor (TNF) receptor superfamily^4^. GPRC5D is an orphan 7-pass transmembrane receptor protein encoded by the *GPRC5D* gene on chromosome 12p^5^. Anti-BCMA or anti-GPRC5D CAR T and TCE therapies have demonstrated promising therapeutic efficacies in relapsed and refractory MM^6–13^. However, the transient durability of clinical response remains an unresolved challenge and the mechanisms underlying immune escape are not fully defined.

Antigenic drift is a well-established tumor-intrinsic mechanism of immunotherapy resistance. The therapeutically enhanced T cell immunity exerts selective pressure on the tumor, which enables the outgrowth of subclones with low or absent target antigens, resulting in tumor immune editing and alterations in the antigenic landscape^14^. Loss of BCMA expression in the MM cells at progression after anti-BCMA CAR T therapy is reported to be rare (3 out of 71; 4%)^9^. Reduction or loss of GPRC5D protein expression was observed in all six cases who progressed after anti-GPRC5D CAR T therapy^11^.

To date, a comprehensive genomic characterization of BCMA loss post-CAR T/TCE therapy has been performed on three patients with BCMA-negative relapse^3,15,16^. In contrast, genomic investigations on intrinsic mechanisms of antigen escape have not been performed in patients who relapsed after anti-GPRC5D CAR T or TCE therapy.

We report, in the present study, a previously, to our knowledge, unrecognized mechanism of BCMA antigenic escape with functional epitope loss secondary to nontruncating mutation and in-frame deletions in the extracellular domain of BCMA. Despite detectable surface BCMA expression, these ectodomain mutations differentially affect the binding affinity and efficacy of anti-BCMA TCEs. We also describe four cases of *GPRC5D* loss after anti-GPRC5D TCE therapy, including two cases with convergent evolution.

## Results

### Study cohort

**Extended Data Fig. 1:**
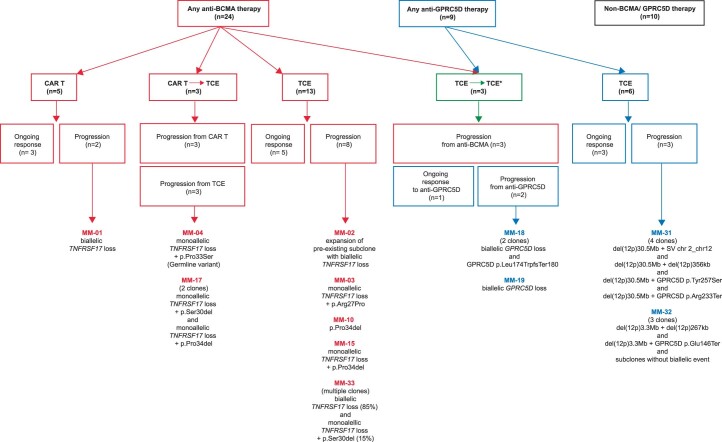
Summary of study patient cohort (n = 40). Diagram illustrates number of patients treated per type of therapy and lists cases with antigen escape in each treatment group. * denotes sequential therapies with anti-BCMA and anti-GPRC5D TCE.

To define the frequency of antigen loss before therapy initiation and at the time of relapse after targeted CAR T or TCE therapy, we assembled a cohort of 40 patients who had relapsed refractory MM (RRMM) treated with anti-BCMA CAR T and/or anti-BCMA and/or anti-GPRC5D TCE therapy, or other non-anti-BCMA/GPRC5D salvage therapies (patients who were anti-BCMA/GPRC5D naive) (Extended Data Fig. 1). The clinical characteristics of each patient at baseline, the types of therapy received (including clinical trial information) and samples analyzed by whole-genome sequencing (WGS) and/or single-cell copy number variation sequencing (scCNV-seq) are summarized in Supplementary Table 1.

Overall, patients received a median of 5 previous lines of therapy (range 2–12) and 30 had triple-class refractory disease. Bone marrow aspirates and/or extramedullary plasmacytoma biopsies were collected before the start of therapy and at the time of progressive disease when feasible. For patient case MM-33, the post-relapse sample was collected 4 months post-progression from TCE therapy (after two additional lines of therapy).

### Biallelic loss of *TNFRSF17* after anti-BCMA CAR T or TCE therapy

Twenty-four patients treated with any anti-BCMA therapy (CAR T, *n* = 5; TCE, *n* = 16; both, *n* = 3) were included in this analysis. Eight patients (33%) had ongoing responses to therapy, whereas 16 (66%) progressed (including 3 patients with primary refractory disease: MM-7, MM-13 and MM-14). All patients with progressive or primary refractory disease had bone marrow samples available for WGS or scCNV-seq or both (Supplementary Table 1). Post-relapse WGS and/or scCNV-seq samples (or pre-therapy samples for patients with primary refractory disease) were available in all 16 patients. Of the 16 patients 8 (50%) had evidence of genomic events on the *TNFRSF17* locus at progression. Of the five patients progressing on anti-BCMA CAR T therapy, only one patient had BCMA loss by biallelic deletion of *TNFRSF17*. Among the 14 patients progressing on anti-BCMA TCE therapy, *TNFRSF17* biallelic loss (*n* = 1) or extracellular domain mutation events (*n* = 5) were detected in 6 patients (42.8%) (Extended Data Fig. 1).

**Fig. 1:**
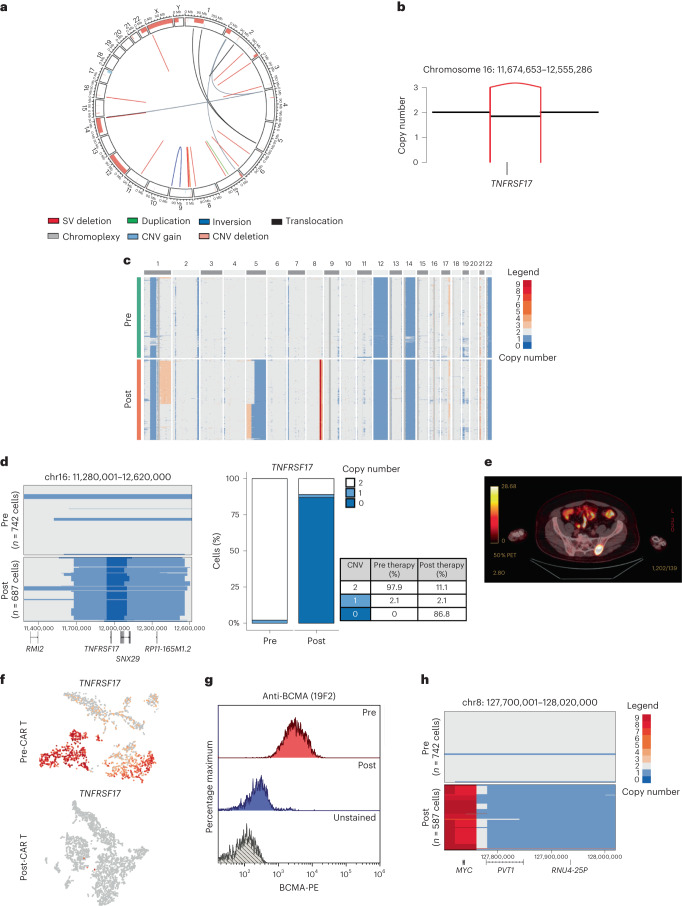
Focal biallelic loss of *TNFRSF17* in patient MM-1 after anti-BCMA CAR T therapy. **a**, Pre-therapy circos plot of patient MM-1 based on WGS. The outer track runs clockwise from chromosome 1 to Y. The inner track shows CNVs (gains in light blue and losses in salmon). The lines inside the circle represent SVs (deletions in red, duplications in green, inversions in blue and interchromosomal translocations in black). **b**, A CN and SV plots showing the subclonal loss of *TNFRSF17* mediated by a focal deletion (red line). **c**, ScCNV-seq heatmap comparing the CN changes in chromosomes 1–22 in pre-therapy (pre) versus post-relapse (post) CD138^+^ MM cells. **d**, Pre-therapy versus post-relapse CD138^+^ CN alteration at *TNFRSF17* locus based on scCNV-seq. The barplot and table compare the percentages of cells harboring the CNVs in pre- versus post-CAR T/TCE relapse samples. **e**, Positron emission tomography scan of patient demonstrating left sacral ala relapse after anti-BCMA CAR T therapy. **f**, Uniform Manifold Approximation and Projection showing the distribution of CD138^+^ cells collected pre- and post-relapse using scRNA-seq. The cells are marked based on the gene expression of *TNFRSF17* (expression in red and no expression in gray). **g**, Pre- versus post-relapse CD138^+^ BCMA protein expression (by monoclonal anti-BCMA antibody (clone 19F2)) using flow cytometry. **h**, CN alterations at MYC locus in pre- versus post-relapse CD138^+^ MM cells based on scCNV-seq.

**Extended Data Fig. 2:**
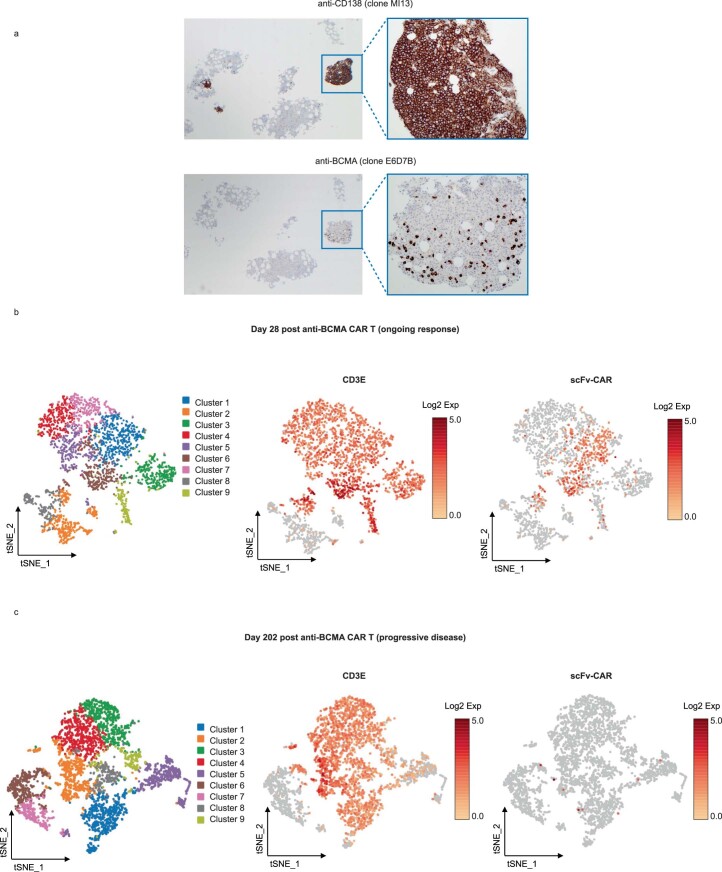
Case MM-01 sacral plasmacytoma and bone marrow biopsy at progressive disease. (**a**) Immunohistochemistry stain of CD138 and BCMA expression on biopsy sample of left sacral ala extramedullary plasmacytoma at relapse. (**b**, **c**) t-distributed stochastic neighbor embedding (tSNE) plot of the distribution of CD3+ cells at pre- (**b**) and post-relapse (**c**) using scRNA. The cells are marked based on the gene expression of CD3ε and scFv-CAR chimeric gene transcript. Pre-sample was collected on day 28 post anti-BCMA CAR T infusion (corresponds to P1800 in Supplementary Table 1) and post-relapse sample was collected on day 202 post CAR T infusion (P1894).

The first case described a patient with RRMM who received idecabtagene vicleucel (Ide-cel) as third-line therapy and relapsed with a BCMA-negative clone (Supplementary Table 1; MM-1). Bulk WGS performed on pre-therapy CD138^+^ cells detected a subclonal structural variant, leading to the deletion of 800 kb encompassing the *TNFRSF17* locus (chr16: 11,674,653–12,555,286) (Fig. 1a,b). ScCNV-seq confirmed a monoallelic *TNFRSF17* deletion in 2.1% of the cells (Fig. 1c,d). At relapse, computed tomography (CT)-guided biopsy of an isolated left sacral ala plasmacytoma (Fig. 1e) confirmed loss of *TNFRSF17* by single-cell RNA-sequencing (scRNA-seq) and flow cytometry (Fig. 1f, g). By scCNV-seq, the subclone with monoallelic loss of *TNFRSF17* before anti-BCMA CAR T exposure accounted for 88.98% of the post-relapse tumor cells. An additional 180-kb focal deletion (chr16: 11,920,001–12,100,000) encompassing the *TNFRSF17* locus was detected at relapse in 86.8% of the cells, resulting in *TNFRSF17* biallelic loss, with multiple copy number (CN) gains at the *MYC* locus (Fig. 1d,h). Of note, 11.1% of clonal plasma cells at progression had no CN alterations at the *TNFRSF17* locus (Fig. 1d and Supplementary Fig. 1) and retained BCMA protein expression (Extended Data Fig. 2a). Last, scRNA-seq analysis of bone marrow CD3^+^ T cells revealed significant CAR T cell contraction at progression (Extended Data Fig. 2b,c).

**Fig. 2:**
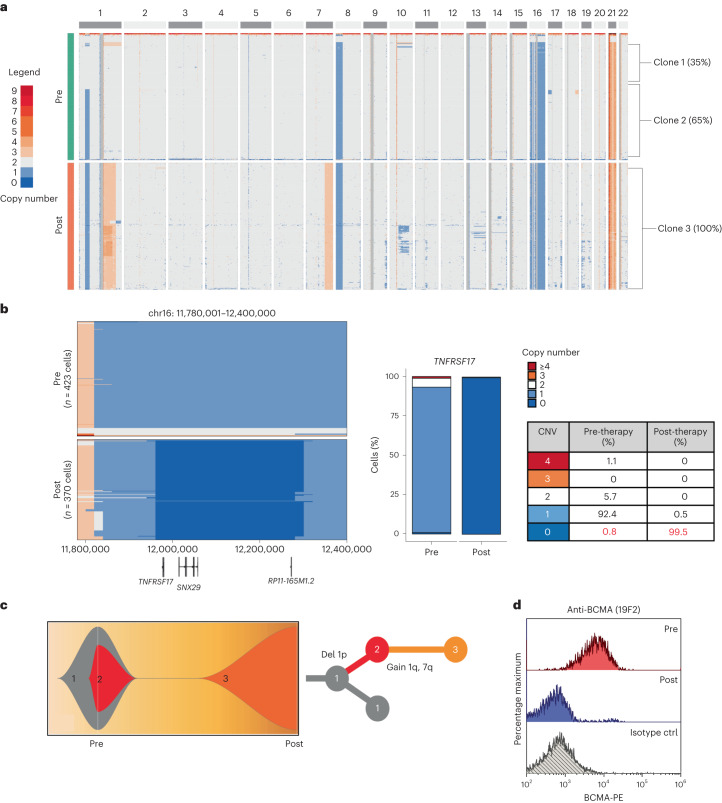
Pre-existing clone with biallelic *TNFRSF17* deletion drives MM relapse after anti-BCMA TCE therapy in patient MM-2. **a**, ScCNV-seq heatmap comparing the CN changes in chromosomes 1–22 in pre-therapy (pre) versus post-relapse (post) CD138^+^ MM cells. **b**, Pre-therapy and post-relapse CD138^+^ CN alteration at *TNFRSF17* locus based on scCNV-seq. The barplot and table compare the percentages of cells harboring the CNVs in pre- versus post-CAR T/ TCE relapse samples. **c**, Fish plot of clonal phylogeny of the CD138^+^ cells at pre-therapy versus post-relapse timepoints inferred from scCNV-seq data. The associated tree illustrates the main alterations differentiating each clone. **d**, Pre- versus post-relapse CD138^+^ BCMA protein expression (by monoclonal anti-BCMA antibody, clone 19F2) using flow cytometry. ctrl, control.

*TNFRSF17* biallelic loss was also identified by scCNV-seq in a patient with triple-class refractory MM (MM-2) who relapsed 6 months after receiving anti-BCMA TCE therapy. The scCNV-seq of CD138^+^ plasma cells at baseline demonstrated a pre-existing monosomy of chromosome 16 in 92.4% of the cells, with subclonal biallelic deletion of *TNFRSF17* in 0.8% of the cells (Fig. 2a,b). ScCNV-seq-inferred clonal phylogeny of pre- and post-relapse samples is shown in Fig. 2c. Consistent with therapy-mediated clonal selection, 99.5% of the cells at relapse harbored biallelic loss of *TNFRSF17* with no BCMA protein expression (Fig. 2b,d).

### BCMA extracellular domain mutations

Anti-BCMA therapy resistance resulting from nontruncating mutations in the BCMA extracellular domain has not, to our knowledge, been previously reported. In the present study, we characterize the impact of four BCMA extracellular domain alterations found in the post-relapse CD138^+^ cells from six patients (Extended Data Fig. 1) who developed resistance to anti-BCMA TCE.

### Nontruncating point mutation in the BCMA extracellular domain

**Fig. 3:**
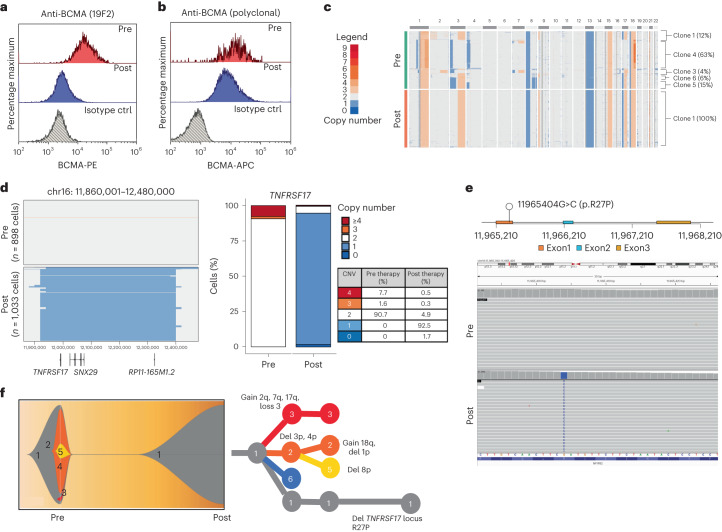
Monoallelic *TNFRSF17* deletion coupled with p.Arg27Pro mutation in the extracellular domain of BCMA mediates MM relapse after anti-BCMA TCE therapy in patient MM-3. **a**, Pre-therapy versus post-relapse CD138^+^ MM cell BCMA protein expression by flow cytometry using monoclonal anti-BCMA antibody (clone 19F2). **b**, Pre-therapy versus post-relapse CD138^+^ BCMA protein expression level by flow cytometry using polyclonal anti-BCMA antibody. **c**, ScCNV-seq heatmap comparing the CN changes in chromosomes 1–22 in pre-therapy (pre) versus post-relapse (post) CD138^+^ MM cells. **d**, Pre-therapy and post-relapse CD138^+^ CN alteration at the *TNFRSF17* locus based on scCNV-seq. The barplot and table compare the percentages of cells harboring the CNVs in pre- versus post-CAR T/TCE relapse samples. **e**, Lollipop plot illustrating the 11965404G>C mutation in exon 1 of the *TNFRSF17* gene. Integrated Genomics Viewer (IGV) screenshot illustrating newly detected clonal point mutation in post-relapse CD138^+^ MM cells. **f**, Fish plot of clonal phylogeny of the CD138^+^ cells at pre-therapy versus post-relapse timepoints inferred from scCNV-seq data. The associated phylogenetic tree illustrates the main alterations differentiating each clone.

Patient MM-3 is a penta-drug refractory MM with no previous exposure to anti-BCMA therapy who received anti-BCMA TCE therapy as the fifth line of treatment with 11 months of complete remission (CR). BCMA expression in pre- versus post-relapse CD138^+^ MM cells using a polyclonal anti-BCMA antibody showed decreased (but detectable) BCMA expression at relapse. In contrast, a monoclonal anti-BCMA antibody (clone 19F2) failed to detect BCMA in the post-relapse sample (Fig. 3a,b).

**Extended Data Fig. 3:**
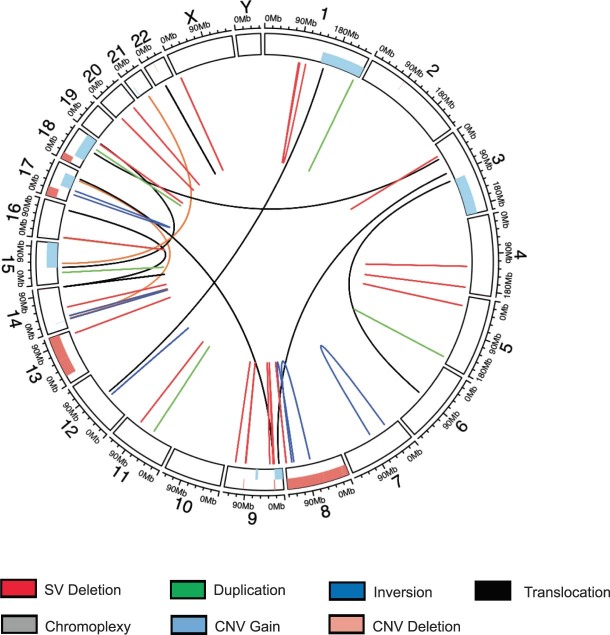
Pre-therapy circos plot of patient MM-03 based on WGS. Outer track runs clockwise from chromosome 1 to Y. Inner track shows CNVs (gains in light blue, losses in salmon). Lines inside the circle represent structural variations (SV) with deletions in red, duplications in green, inversions in blue, interchromosomal translocations in black, and chromoplexy in grey.

ScCNV-seq and bulk WGS on bone marrow CD138^+^ MM cells at relapse demonstrated a monoallelic focal loss of *TNFRSF17* coupled with a clonal missense mutation in exon 1 of *TNFRSF17* c.80G>C (p.Arg27Pro) (Fig. 3c–e). Clonal phylogeny was inferred from the scCNV-seq data (Fig. 3f). Neither the deletion nor the mutation of *TNFRSF17* was detectable in the baseline samples by scCNV-seq, WGS (100×) or digital PCR (dPCR; limit of detection of 0.1% allelic frequency) (Extended Data Fig. 3 and Supplementary Fig. 2a,d).

**Fig. 4:**
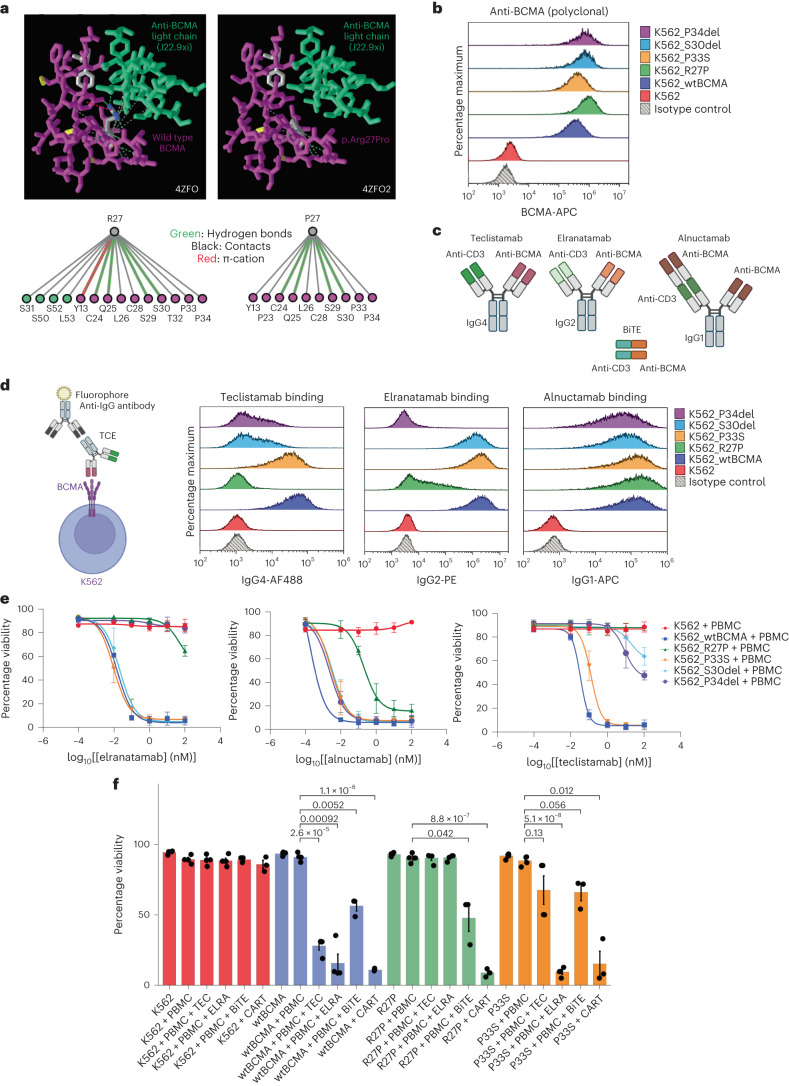
Mutation in the BCMA extracellular domain abrogates TCE binding and TCE-mediated target cell death. **a**, Publicly deposited crystal structure (PDB, accession no. 4ZFO) by Marino et al.^18^ representing the interaction between wild-type or p.Arg27Pro BCMA and J22.9-xi light chain. It was analyzed using the Macromolecular Structures Resource Group^20^. **b**, BCMA protein expression in the established K562 cell lines using polyclonal anti-BCMA antibodies by flow cytometry. **c**, Illustration of the structures of anti-BCMAxCD3ε TCEs screened in the present study. The figure was created with BioRender. **d**, TCE-binding assay. The figure was created with BioRender. K562 cell lines were incubated with teclistamab, elranatamab or alnuctamab (10 nM) followed by secondary anti-IgG flow antibody staining. **e**, TCE dose response curve (DRC). K562 target cell viability 48 h after co-culture with healthy donor PBMCs at an effector:target ratio of 10:1. K562 cells were pre-stained with CTV and cell viability was assessed by staining with calcein AM and PI for flow cytometry assessment. The TCE doses range from 0.01 nM to 100 nM. Data are presented as mean ± s.d. The samples are biologically independent (*n* = 3 for all cell lines). **f**, Barplot showing percentage viability of K562 48 h after co-culture with healthy donor PBCMs at an effector:target ratio of 10:1 with or without the respective TCEs (0.1 nM) or BiTE (5.4 nM) as indicated. Co-culture with anti-BCMA CAR T was performed at an effector:target ratio of 1:1. The *x* axis corresponds to the experimental conditions. The samples shown are biologically independent for K562 alone (*n* = 4), K562 + PBMCs (*n* = 4), K562 + PBMCs + TEC (*n* = 4), K562 + PBMCs + ERLA (*n* = 4), K562 + PBMCs + BiTE (*n* = 3) and K562 + PBMCs + CAR T (*n* = 3). The Student’s *t*-test was performed on each grouped sample (each K562 cell line) without adjustments for multiple comparisons using the R function pairwise.t.test() to generate *P* values. Absolute *P* values are provided in Supplementary Table 8. Data are presented as mean ± s.d. Source data

BCMA Arg27 interacts with the complementarity-determining regions (CDRs) of the heavy chain of the anti-BCMA variable region of teclistamab^17^. Arg27 is also involved in forming contacts with a chimeric mouse/human anti-BCMA antibody (J22.9-xi) light chain^18^. Modeling of wild-type versus p.Arg27Pro mutant BCMA, using the publicly deposited crystal structure representing the interaction between BCMA and J22.9-xi, demonstrated that p.Arg27Pro disrupts all contacts between the BCMA and the light chain of J22.9-xi (Fig. 4a) (Protein Data Bank (PDB), accession no. 4ZFO)^18–20^.

**Extended Data Fig. 4:**
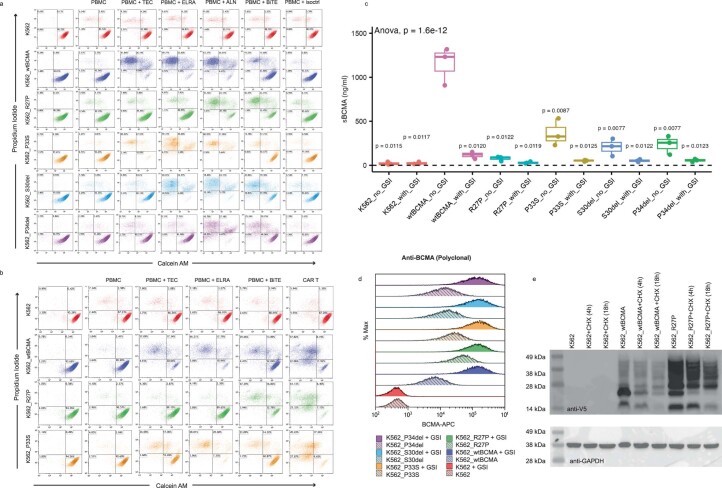
BCMA wild type and mutant K562 cell cytotoxicity assay, soluble BCMA, and protein stability assay. K562 target cells were pre-stained with CellTrace Violet (CTV) prior to co-culture. Panels (**a**) and (**b**) represent representative cytotoxicity assays flow cytometry contour with density plots gated on CTV positive cells. In panel (a), target cells were co-cultured with healthy donor PBMCs at 10:1 effector to target (E:T) ratio ± Teclistamab 1 nM (TEC), Elranatamab 1 nM (ELRA), Alnuctamab 1 nM (ALN), anti-BCMAxCD3 BPS 5.4 nM (BiTE), or isotype control antibody 1 nM (isoctrl). In panel (b), target cells were co-cultured with healthy donor PBMC (E:T = 10:1) ± TCE at 0.1 nM doses or co-cultured with or anti-BCMA CAR T (E:T = 1:1). (**c**) Soluble BCMA measurement from the supernatant of K562 cell line cultures using sBCMA DuoSet kit. The cell lines were cultured at 1 × 10^6^ cells/ mL ± 6.2 nM gamma secretase inhibitor (GSI) overnight. P-values were generated by t-test comparing each condition to wtBCMA_no_GSI using the R function pairwise.t.test() without adjustments for multiple comparisons. Box plot illustrates median (horizontal line through box), upper hinge (75^th^ percentile, Q3), lower hinge (25^th^ percentile, Q1). Upper hinge extends to Q3 + 1.5 x interquartile range (IQR). Lower hinge whisker extends to Q1-1.5 x IQR. N = 3 biologically independent samples. (**d**) Surface BCMA expression in K562 or U266 cell lines by polyclonal anti-BCMA antibody by flow cytometry. Cells were treated with or without 6.2 nM GSI overnight. (**e**) Western blot of cycloheximide chase experiment for estimation of BCMA protein stability. K562 cell lines were treated with or without 10 ug/mL of cycloheximide (CHX) for the indicated times. Cell lysates were collected for western blot for V5 tagged BCMA and GAPDH. Source data

To evaluate the effect of p.Arg27Pro on the efficacies of various anti-BCMA TCE therapies, we transduced the K562 myelogenous cell line to express either wild-type (K562_wtBCMA) or p.Arg27Pro BCMA (K562_R27P) (Fig. 4b). Teclistamab and elranatamab are symmetrical bispecific antibodies and contain one BCMA-binding Fab domain with an immunoglobulin (Ig)G4 or IgG2 Fc backbone, respectively. Alnuctamab has two anti-BCMA Fab molecules in asymmetrical design with an IgG1 Fc backbone (Fig. 4c). We screened the binding efficacies and cytotoxic effect of these TCEs. All three TCEs were bound to K562_wtBCMA, whereas only alnuctamab was detectable on the surface of the K562_R27P cells (Fig. 4d). K562_R27P clones also exhibited differential TCE sensitivity with resistance to teclistamab- and elranatamab-mediated cytotoxicity, while retaining sensitivity to alnuctamab and a commercially available anti-BCMAxCD3 tandem scFv, BiTE (BPS Bioscience) (Fig. 4e and Extended Data Fig. 4a). Notably, K562_R27P was also sensitive to an in-house manufactured anti-BCMA CAR T derived from Ide-cel (Fig. 4f and Extended Data Fig. 4b)^21^.

Not all identified BCMA extracellular domain missense mutations impacted the efficacy of anti-BCMA T cell therapies. A BCMA germline variant (p.Pro33Ser) was identified in patient MM-4 who had minimal disease response to Ide-cel and had primary refractory disease to anti-BCMA TCE in the subsequent line of therapy (Supplementary Fig. 3). Despite the clinical behavior observed in this patient, K562 cells expressing p.Pro33Ser mutant BCMA (K562_P33S) demonstrated binding of all three TCEs (Fig. 4d) and were sensitive to anti-BCMA TCEs and CAR T-mediated cytotoxicity^21^ (Fig. 4e,f and Extended Data Fig. 4a,b).

### In-frame deletions in BCMA extracellular domain

In-frame deletions in the BCMA ectodomain, including Pro34 deletion (p.Pro34del, *n* = 2 patients) or Ser30 deletion (p.Ser30del, *n* = 1 patient) or both deletions (*n* = 1 patient) were identified in four patients at relapse after anti-BCMA TCE therapy (Extended Data Fig. 1).

**Fig. 5:**
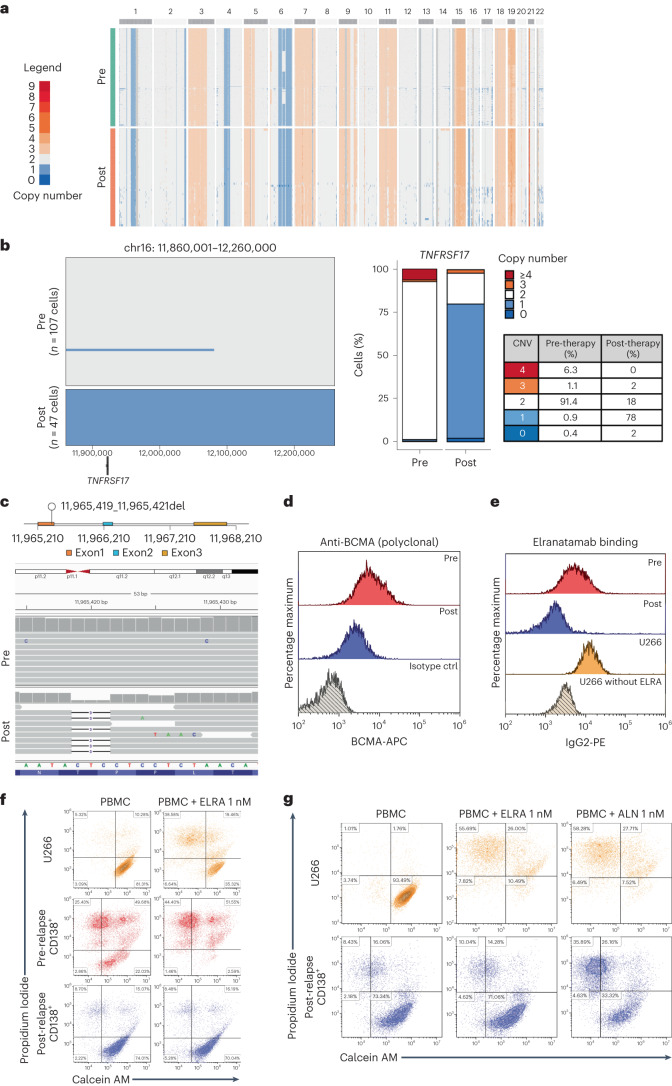
Monoallelic *TNFRSF17* deletion coupled with deletion of Pro34 in the BCMA extracellular domain mediates MM relapse after anti-BCMA TCE therapy in patient MM-15. **a,** ScCNV-seq heatmap comparing the CN changes in chromosomes 1–22 in pre-therapy (pre) versus post-relapse (post) CD138^+^ MM cells. **b**, Pre-therapy and post-relapse CD138^+^ CN alteration at *TNFRSF17* locus based on scCNV-seq. The barplot and table compare the percentages of cells harboring the CNVs in pre- versus post-CAR T/TCE relapse samples. **c**, Lollipop plot illustrating the 11965419_11965421del in exon 1 of *TNFRSF17* gene. The IGV screenshot illustrates newly detected clonal point mutations in post-relapse CD138^+^ MM cells. Deletion of 3 bp (chr16: g.11,965,419–11,965,421del) in exon 1 of *TNFRSF17* was detected in the post-relapse CD138^+^ MM sample. This in-frame deletion removes the last two nucleotides of the Thr32 codon and the first nucleotide of the Pro33 codon. This results in retention of Thr32 but deletion of one of the two consecutive proline residues. **d**, Pre- versus post-relapse CD138^+^ BCMA protein expression (by polyclonal anti-BCMA antibody) using flow cytometry. **e**, Pre- or post-relapse patient primary CD138^+^ cells or U266 MM cell lines incubated with elranatamab (1 nM) followed by secondary flow antibody staining with anti-IgG2 flow antibody. **f**, Flow cytometry contour with density plot, gated on CTV-positive cells. Patient MM-15’s pre- versus post-relapse CD138^+^ MM cells or U266 cell line was pre-stained with CTV and co-cultured with patient autologous PBMCs at an effector:target ratio of 10:1, with or without elranatamab (1 nM). The target cell viability is shown at 24 h after co-culture. **g,** Flow cytometry contour with density plot, gated on CTV-positive cells. Patient’s post-relapse CD138^+^ MM cell and U266 cell line viability shown at 48 h after co-culture with autologous PBMCs (effector:target = 10:1) with elranatamab 1 nM or alnuctamab 1 nM.

Patient MM-15 attained stringent CR after anti-BCMA TCE therapy lasting 12 months. ScCNV-seq of pre- versus post-relapse CD138^+^ samples demonstrated monoallelic loss of chromosome 16p (Fig. 5a,b), in addition to a three-nucleotide deletion (c.98_100del) that leads to an in-frame deletion of Pro34 (p.Pro34del) (Fig. 5c). BCMA surface expression as well as elranatamab binding on CD138^+^ cells were verified by flow cytometry, demonstrating loss of engagement of the TCE on the post-relapse CD138^+^ cells (Fig. 5d,e) and resistance to elranatamab compared with the pre-CD138^+^ cells and the MM U266 cell line (Fig. 5f). Of interest, post-relapse CD138^+^ cells retained their sensitivity to alnuctamab (Fig. 5g). Consistent results were also obtained in co-culture experiments in K562 clones expressing p.Pro34del BCMA (K562_P34del), with resistance to teclistamab and elranatamab, but not to alnuctamab (Fig. 4e and Extended Data Fig. 4a).

**Extended Data Fig. 5:**
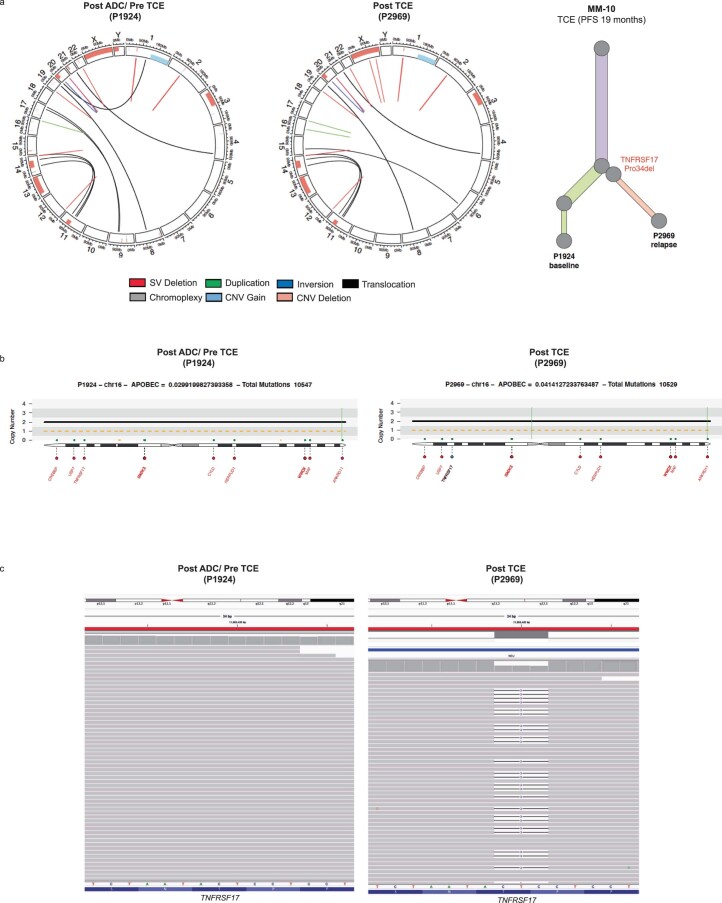
Case MM-10 demonstrates p.Pro34del MM clone at relapse. (**a**) Pre-therapy (pre ADC and pre TCE, sample number P1924) versus post-relapse (post TCE, P2969) circos plot of patient MM-10 based on WGS. Outer track runs clockwise from chromosome 1 to Y. Inner track shows CNVs (gains in light blue, losses in salmon). Lines inside the circle represent SVs (deletions in red, duplications in green, inversions in blue, interchromosomal translocations in black, and chromoplexy in grey). Clonal phylogeny inferred from WGS data. (**b**) Copy number and structural variations at *TNFRSF17* locus. Translocations are represented in black, deletions in red, duplications in green, and inversions in blue. (**c**) IGV screenshot of pre-therapy versus post-relapse samples, illustrating newly detected p.Pro34del in post-relapse CD138 + MM cells.

Clonal BCMA p.Pro34del was identified in another case at relapse (MM-10) who received anti-BCMA TCE as the sixth line of therapy lasting 19 months. Compared with pre-anti-BCMA TCE, CD138^+^ MM cells at relapse harbored a clonal 3-nucleotide deletion (*TNFRSF17* c.98_100del, variant allele fraction (VAF) of 100% corrected for sample purity (45%) by allele-specific CN analysis of tumors (ASCAT) and Battenberg). No CN loss at *TNFRSF17* locus was observed (Extended Data Fig. 5a–d).

A second in-frame deletion on *TNFRSF17*, p.Ser30del, was identified at relapse in patient MM-33 after attaining stringent CR after anti-BCMA TCE therapy. Biallelic deletions encompassing *TNFRSF17* locus were identified in 85% of the cells whereas a subclonal fraction (15% cells) harbored in-frame deletion generating a BCMA variant lacking one of the consecutive serine residues at positions 29 and 30 of BCMA (p.Ser30del) (Supplementary Fig. 4a,b). Functionally, K562 cells expressing p.Ser30del (K562_S30del) were resistant to teclistamab but retained sensitivity to elranatamab and alnuctamab (Fig. 4e and Extended Data Fig. 4a).

**Extended Data Fig. 6:**
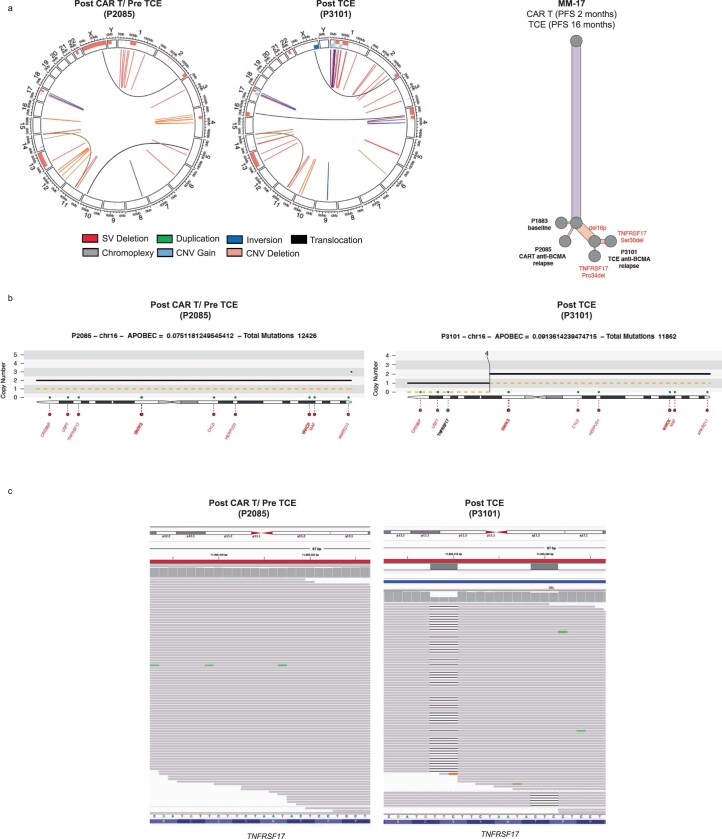
Newly detected p.Pro34del and p.Ser30del BCMA in MM clones at relapse in case MM-17. (**a**) Pre-therapy (post CAR T and pre TCE, sample number P2085) versus post-relapse (post TCE, P3101) circos plot based on WGS. Outer track runs clockwise from chromosome 1 to Y. Inner track shows CNVs (gains in light blue, losses in salmon). Lines inside the circle represent SVs (deletions in red, duplications in green, inversions in blue, interchromosomal translocations in black, and chromoplexy in grey). Clonal phylogeny inferred from WGS data. (**b**) Copy number and structural variations at *TNFRSF17* locus. Translocations in black, deletions in red, duplications in green, inversions in blue. (**c**) IGV screenshot of pre- versus post-relapse CD138+ cells, illustrating newly detected p.Pro34del and p.Ser30del at relapse.

Notably, patient MM-17 showed evidence of convergent evolution with two independent subclones each harboring *TNFRSF17* extracellular domain SNVs coupled with monoallelic *TNFRSF17* loss at relapse after anti-BCMA TCE therapy. This patient received Ide-cel as the third line of therapy resulting in 2 months of stable disease. Subsequent therapy with anti-BCMA TCE resulted in 16 months of stringent CR before relapse. Serial bone marrow CD138^+^ samples from pre-CAR T, post-CAR T/pre-TCE and post-TCE timepoints demonstrated the emergence of a major subclone with p.Ser30del (c.87_89del) VAF of 66.5% corrected for sample purity (83% by ASCAT and Battenberg) and a minor subclone with p.Pro34del (c.98_100del, VAF 10%), along with monoallelic *TNFRSF17* deletion at relapse post-TCE therapy (Extended Data Fig. 6a–d). Neither of the *TNFRSF17* in-frame deletions was detected at baseline by dPCR (Supplementary Fig. 2b,c)

Computational modeling of BCMA demonstrated that all mutations described in the present study induce rigidification and conformational change of BCMA (Supplementary Fig. 5a-c)^22^, altering its binding interface between anti-BCMA TCEs (Supplementary Fig. 6a,b).

### BCMA extracellular domain mutations affect soluble BCMA

γ-Secretases release the extracellular domain of BCMA-generating soluble peptide (sBCMA)^23^. Significantly higher levels of sBCMA were detected (by polyclonal anti-BCMA antibodies) from the supernatant of K562_wtBCMA cells compared with K562 BCMA mutant cells (Extended Data Fig. 4c,d). In the cycloheximide chase assay, we observed no difference in protein stability of p.Arg27Pro BCMA compared with the wild-type (Extended Data Fig. 4e). Altogether, these data suggest that selected mutations in the BCMA ectodomain interfere with γ-secretase cleavage, generating lower sBCMA levels discordant with tumor burden.

### APRIL ligand binding and NF-κB activation

**Extended Data Fig. 7:**
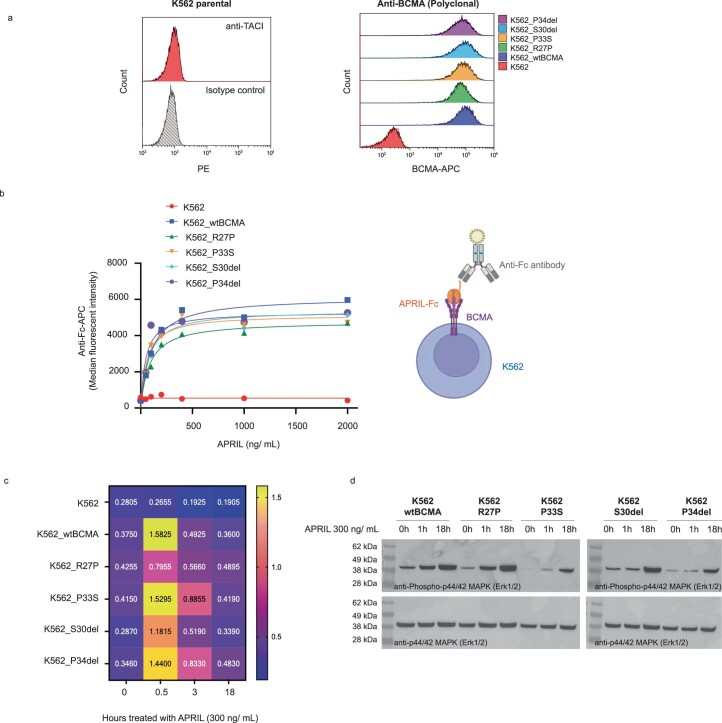
APRIL binding and NFkB signaling in BCMA wild type and mutant K562 cell lines. (**a**) First histogram shows TACI expression in K562 cell line and second panel shows transgenic BCMA expression in K562 cell lines by flow cytometry. (**b**) Dose response curve for APRIL binding. K562 cell lines expressing wild type or mutant BCMA were incubated with Fc tagged APRIL trimer at indicated doses followed by anti-Fc APC flow antibody staining. (**c**) NFkB p65 ELISA was performed using nuclear extracts from K562 cell lines treated with APRIL (300 ng/ mL) for the indicated time points. (**d**) Western blot for phosphorylated and total ERK was performed using total cell lysates from K562 cell lines treated with APRIL (300 ng/ mL) for indicated time points. Source data

We assessed whether BCMA ectodomain mutations affect APRIL (a proliferation-inducing ligand) binding and downstream nuclear factor κ-light-chain-enhancer of activated B cells (NF-κB) signaling in parental K562 cells (lacking transmembrane activator and CAML interactor (TACI)) as well as transduced K562 cells expressing wild-type or mutant BCMA (Extended Data Fig. 7a). Flow cytometry using anti-Fc antibodies demonstrated that Fc-tagged APRIL trimer did bind wild-type and mutant BCMA-expressing K562 cells (Extended Data Fig. 7b) and equally activated NF-κB signaling (p65 ELISA and western blotting for phosphorylated ERK (extracellular signal-regulated kinase)) (Extended Data Fig. 7c,d).

Finally, we observed a nonsignificant trend of NF-κB pathway-activating mutagenic events in patients with *TNFRSF17* biallelic loss or mutations, with three out of the six patients harboring clonal *TRAF3* or *CYLD* or *MAP3K14* biallelic deletion or SNVs (Supplementary Fig. 7).

### *GPRC5D* biallelic deletion post anti-GPRC5D TCE therapy

**Fig. 6:**
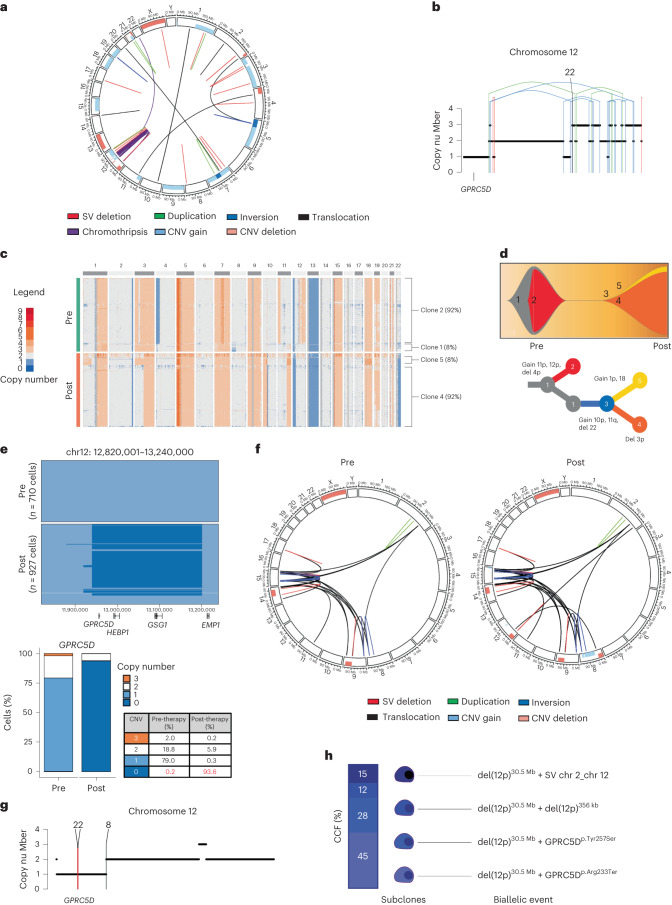
*GPRC5D* biallelic loss mediates MM relapse after anti-GPRC5D TCE therapy. **a**, Pre-therapy circos plot of patient MM-18 based on WGS. The outer track runs clockwise from chromosome 1 to Y. The inner track shows CNVs (gains in light blue and losses in salmon). The lines inside the circle represent SVs (deletions in red, duplications in green, inversions in blue and interchromosomal translocations in black). **b**, Illustration of chromosome 12 with CN loss of *GPRC5D* mediated by a chromothripsis event (translocations in black, deletions in red, duplications in green and inversions in blue) (patient MM-18). **c**, ScCNV-seq heatmap comparing the CN changes in chromosomes 1–22 in pre-therapy (pre) versus post-relapse (post) CD138^+^ MM cells (patient MM-18). **d**, Fish plot of clonal phylogeny of the CD138^+^ cells at pre-therapy versus post-relapse timepoints inferred from scCNV-seq data. The associated tree illustrates the main alterations differentiating each clone (patient MM-18). **e**, Pre-therapy versus post-relapse CD138^+^ CN alteration at the *GPRC5D* locus based on scCNV-seq. The barplot and table compare the percentages of cells harboring the CNVs in pre- versus post-TCE relapse samples (patient MM-18). **f**, MM-31 circos plot of WGS data with CNVs and SVs pre-talquetamab treatment and at relapse post-talquetamab treatment. The outer track runs clockwise from chromosome 1 to Y. The inner track shows CNVs (gains in light blue and losses in salmon). The lines inside the circle represent SVs (deletions in red, duplications in green, inversions in blue and interchromosomal translocations in black). **g**, Illustration of chromosome 12 CNVs and SVs in *GPRC5D* from WGS (patient MM-31). **h**, Illustration of cancer cell fractions and subclonal events in the short arm of chromosome 12, resulting in biallelic *GPRC5D* alteration at relapse (patient MM-31).

**Extended Data Fig. 8:**
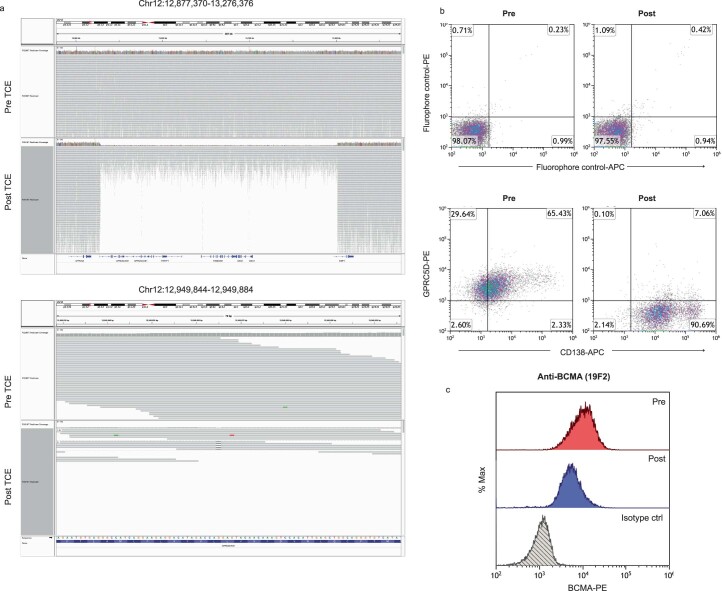
Case MM-18 WGS and flow cytometry analysis. (**a**) IGV screenshot of pre- versus post-relapse CD138+ cells sorted by insert size showing deletions of 272 kilobases (chr12:12,928,352-13,200,487) at *GPRC5D* locus on chromosome 12 (top panel) based on WGS. Bottom panel depicting residual reads with frameshift deletion in 5 out of 11 reads (chr12: g.12,949,864delG) resulting in early stop codon and truncation of GPRC5D (p.Leu174TrpfsTer180). (**b**, **c**) Pre- versus post-relapse CD138+ cell flow cytometry analysis demonstrating (b) GPRC5D and (c) BCMA protein expression.

We have reported four cases of GPRC5D-negative relapses after anti-GPRC5D TCE therapy in the present study. MM-18 was treated with talquetamab, daratumumab, pomalidomide and dexamethasone (Talq-DPd) as the sixth line of therapy achieving 1 year of remission. This patient had received teclistamab with stable disease and progression-free survival (PFS) of 2 months immediately before receiving talquetamab. Bulk WGS on the pre-therapy sample revealed evidence of chromothripsis on chromosome 12, along with a pre-existing one CN loss of *GPRC5D* (Fig. 6a,b). ScCNV-seq analysis on CD138^+^ cells pre-talquetamab demonstrated monoallelic (79%) and biallelic (0.2%) loss of *GPRC5D*, whereas, at relapse, clonal biallelic *GPRC5D* focal deletion of 220 kb was seen in 93.6% of cells (Fig. 6c–e). WGS confirmed *GPRC5D* biallelic deletions in 90% of the cells whereas a subclonal fraction (5%) harbored a GPRC5D p.Leu174TrpfsTer180 frameshift deletion, resulting in an early stop codon (Extended Data Fig. 8a). Flow cytometry analysis confirmed loss of GPRC5D expression (Extended Data Fig. 8b,c).

**Extended Data Fig. 9:**
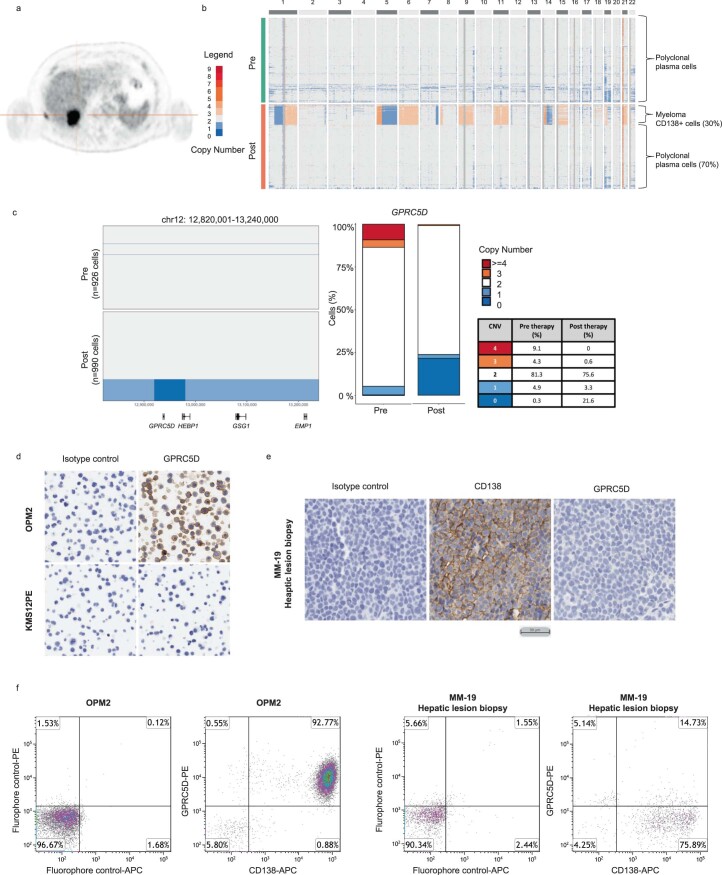
*GPRC5D* biallelic loss mediates MM relapse post anti-GPRC5D TCE in case MM-19. (**a**) Positron emission tomography scan image of hepatic extramedullary relapse post anti-GPRC5D TCE. (**b**) scCNV heatmap comparing the copy number changes in chromosomes 1-22 in pre-therapy (pre) versus post-relapse (post) CD138 + MM cells. Bone marrow CD138+ cells were predominantly enriched with normal polyclonal plasma cells as evidenced by diploid population. (**c**) Pre-therapy versus post-relapse CD138+ copy number alteration at *GPRC5D* locus based on scCNV. Barplot and table comparing the percentages of cells harboring the CNV in pre- versus post-TCE relapse samples. (**d**, **e**) Immunohistochemistry (IHC) staining of CD138 and GPCR5D expression on (d) control cell lines OPM2 (positive control) and KMS12PE (negative control) and (e) liver biopsy sample from patient. (**f**) Flow cytometry assay of CD138 and GPRC5D expression on OPM2 (positive control) and liver biopsy sample from patient. Post relapse CD138+ cell are negative for GPRC5D by both IHC and flow cytometry.

Patient MM-19, who had extramedullary hepatic disease treated with Talq-DPd, achieved stringent CR lasting 15 months (Extended Data Fig. 9a). ScCNV-seq analysis of clonal (30%) bone marrow plasma cells at relapse demonstrated a biallelic loss of *GPRC5D* (Extended Data Fig. 9a–c). Immunohistochemistry (IHC) staining and flow cytometry of CD138^+^ cells from another biopsy of an extramedullary hepatic plasmacytoma after subsequent anti-BCMA TCE therapy, confirmed *GPRC5D* loss (Extended Data Fig. 9d–f).

**Extended Data Fig. 10:**
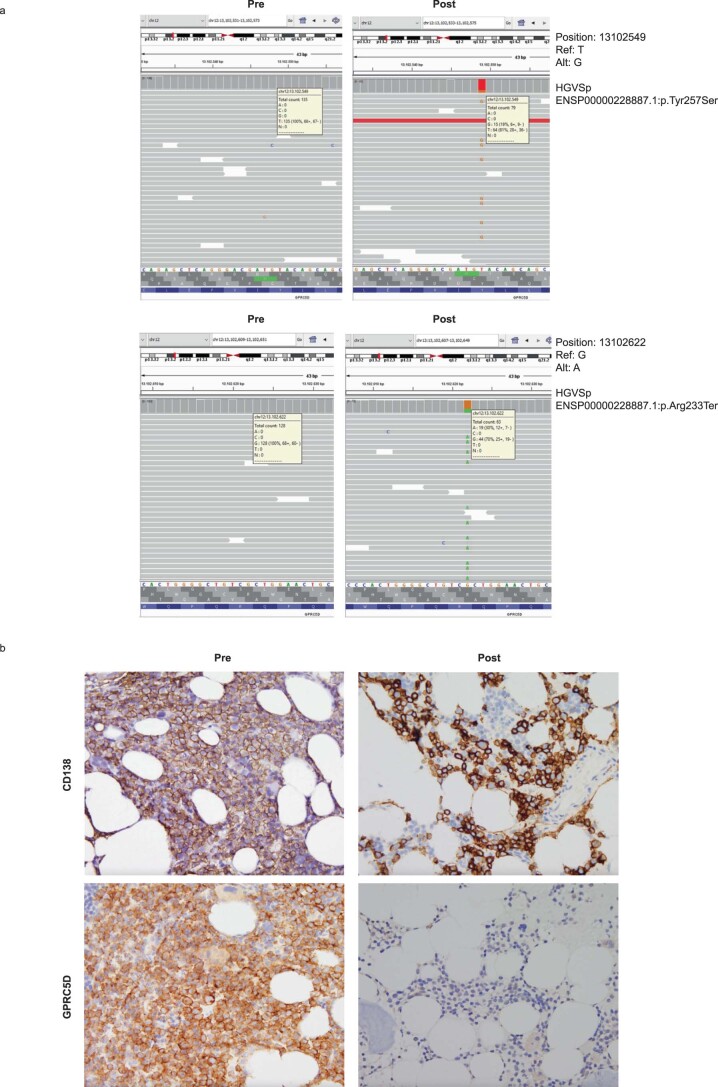
Case MM-31 demonstrates GPRC5D antigen escape post anti-GPRC5D TCE. (**a**) IGV screenshot of the pre- versus post-relapse CD138+ cells demonstrating that mutations in *GPRC5D* (structural variations, copy number variations, single nucleotide variations) were not detected prior to therapy. (**b**) Immunohistochemistry staining of CD138 and GPRC5D expression in the pre- versus post-relapse bone marrow biopsy samples.

Patient MM-31 received talquetamab as the seventh line of therapy. Baseline WGS analysis of the CD138^+^ cells collected 1 year before the start of talquetamab showed no biallelic or monoallelic alteration of *GPRC5D* (Fig. 6f and Extended Data Fig. 10a). At relapse (15 months after the initiation of therapy), CD138^+^ MM cells demonstrated a large deletion (30.5 Mb) in the short arm of chromosome 12, encompassing the *GPRC5D* gene locus (Fig. 6g). Notably, WGS revealed a complex subclonal structure consistent with convergent evolution toward biallelic *GPRC5D* loss. Although around 45% of MM cells harbored a nonsense mutation (p.Arg233Ter), a missense mutation in *GPRC5D* (p.Tyr257Ser) with a cancer cell fraction (CCF) of 28% represented another subclone because both mutations were mutually exclusive after phasing the reads. WGS data also indicated an additional small subclone (CCF 12%) with a biallelic deletion of 356 kb (chr12: 13,002,001–13,358,000; hg19 reference genome) within the monoallelic chromosome 12p deletion leading to biallelic *GPRC5D* loss. A fourth subclone (CCF 15%) showed biallelic loss of *GPRC5D* (from 13,001,208 to 13,356,723) with a reciprocal translocation involving the long arm of chromosome 2 and the short arm of chromosome 12 (Fig. 6h). IHC staining of pre- versus post-relapse bone marrow biopsy samples confirmed loss of *GPRC5D* (Extended Data Fig. 10b). This case shows emergence of a clone with chromosome 12p deletion and convergent evolution of several subclones with biallelic *GPRC5D* inactivation caused by variable genomic events.

Similarly, MM-32 progressed with *GPRC5D* loss after anti-*GPRC5D* TCE therapy and showed a large chromosome 12p deletion of 3.3 Mb and two subclones with aberrations affecting *GPRC5D* (Supplementary Fig. 8a). One subclone harbored a mutation in *GPRC5D* (p.Glu146Ter, CCF 35%) and a second subclone with biallelic *GPRC5D* loss caused by a biallelic deletion of 267 kb (chr12: 130,150,01–132,820,00) in 7% of the CD138^+^ cells. However, a remaining fraction of 58% of the cells did not show a biallelic event in *GPRC5D* (Supplementary Fig. 8b), suggesting that additional mechanisms may be mediating resistance.

### Pre-existing focal *TNFRSF17* and *GPRC5D* loss

#### WGS analysis

To define the prevalence of *TNFRSF17* and *GPRC5D* subclonal mutations, we interrogated WGS data of 44 samples (28 pre- and 16 post-CAR T/TCE samples) collected from 31 patients (median 100× coverage) (Supplementary Table 1). By WGS, two patients (MM-1 and MM-2) had subclonal and clonal single CN loss, respectively, of the *TNFRSF17* gene locus at baseline (2 out of 28: 7.14%) (Fig. 1a,b and Supplementary Fig. 9a,b). Of interest, both patients relapsed with biallelic deletion of *TNFRSF17*. With respect to *GPRC5D*, monoallelic large and focal deletions were noted in six patients who were GPRC5D CAR T/TCE naive (6 out of 28: 21.4%) (Supplementary Fig. 10).

Analysis of the CoMMpass dataset (*n* = 896 patients) revealed, at baseline, monoallelic loss of *TNFRSF17* and *GPRC5D* in 3.35% and 13.17%, respectively (Supplementary Fig. 11)^24^. Furthermore, 11 samples were found to have mutational events on *TNFSRF17* (1.1%) (Supplementary Table 3), all restricted to the transmembrane and intracellular domains of BCMA. Nine of these were internal tandem repeats, one a frameshift and one a nonsense variant^24^.

Last, interrogating the MLL Munich Leukemia Laboratory WGS dataset of 4,995 patients with various myeloid and lymphoid neoplasms (including 20 patients with monoclonal gammopathy of undetermined significance and 399 MM patients with anti-BCMA therapy-naive patients), 33 patients (0.7%, including 3 MM patients) demonstrated germline variants or variants of unknown significance in *TNFRSF17*. The list of identified mutations is given in Supplementary Table 4. In 15 patients, the missense variant affected the extracellular domain of BCMA. The germline variant identified in patient MM-4, p.Pro33Ser, was found in four patients. Other variants that occurred in more than one patient were p.Ala54Thr (*n* = 5), pCys59Tyr (*n* = 2), p.Pro145Ser (*n* = 2) and p.Met74del (*n* = 2). Of interest, the missense mutation p.Arg27Pro and the two in-frame deletions p.Pro34del and p.Ser30del were not found as germline or somatic variants in this dataset.

### scCNV-seq analysis

Among 24,643 MM cells analyzed by scCNV-seq, in patients who were CAR T/TCE therapy naive (*n* = 15 patients, 26 pre- and 9 post-CAR T/TCE samples), monoallelic or biallelic CN loss of *TNFRSF17* was detected in 1.8% and 0.3% of the cells, respectively, whereas 3 and 4 or more CN gains were noted in 1.9% and 6% of the cells, respectively (Supplementary Table 5a,b and Supplementary Fig. 12a). Among patients progressing on anti-BCMA CAR T/TCE therapy (*n* = 8 patients, 6,464 cells), *TNFRSF17* gain or mono- or biallelic loss was detected in 6.4%, 18% and 21.2% of cells, respectively (Supplementary Table 5a,c). In the same cohort, *GPRC5D* monoallelic or biallelic loss was seen in 0.2% and 3.9% of the cells, respectively.

Comparison of CNVs in loci of other myeloma-relevant genes in CAR T/TCE naive (*n* = 15 patients, 26 samples) versus CAR T/TCE relapse cases (*n* = 10 patients, 10 samples) is summarized in Supplementary Table 6 (Supplementary Fig. 12b). Notably, single-cell CN gains at the *FCRL5* and *SLAMF7* loci on chromosome 1q were observed in relapsed samples. Last, in two patients (MM-18 and MM-20) who progressed on anti-BCMA TCE with no *TNFRSF17* loss or ectodomain mutations, CN gain (≥3 copies) at *TNFRSF17* locus was noted at relapse (19.9% and 20.4%, respectively) (Supplementary Table 5c).

## Discussion

The emergence of BCMA mutants after immunoselection by targeted CAR T and TCE therapy is an important, albeit previously considered rare, mechanism of MM resistance to BCMA-targeting immunotherapies. Three published cases on genomic loss of BCMA have been reported to date^3,15,16^, all of which share a similar mechanism of biallelic *TNFRSF17* loss involving a large pre-existing chromosome 16p loss, followed by a second focal genomic hit on the remaining allele. Based on these observations, it was postulated that monoallelic 16p loss may represent a genomic feature associated with higher risk of progression after anti-BCMA CAR T/TCE therapy. Different to these cases, the first case of *TNFRSF17* biallelic loss (MM-1) in our study involved clonal focal events at both *TNFRSF17* loci without large-scale aberrations or monosomy of chromosome 16. This suggests that large structural events on chromosome 16 are not the only predisposing risk factors for BCMA-negative relapse, and focal biallelic deletions of *TNFRSF17* can occur even in the setting of diploid chromosome 16p.

After Ide-cel therapy, rare biallelic loss of *TNFRSF17* was reported at relapse (6%) suggesting that BCMA antigenic loss may not be the main driver of resistance^25^. In contrast, after anti-BCMA TCE, we observed a higher rate of BCMA mutational events than previously perceived with diverse mechanisms leading to antigenic loss. In our series, mutational events in *TNFRSF17* were identified in 6 out of 14 patients (42.8%) who experienced disease progression after anti-BCMA TCE. This difference in the rates of mutational events involving *TNFRSF17* gene locus may stem from the longitudinal selective therapeutic pressure with TCE in comparison to the transient immune selection post-CAR T.

We present previously undescribed cases of *TNFRSF17* extracellular domain mutations mediating loss of functional BCMA epitopes. The clonal p.Arg27Pro missense mutation, or in-frame deletions of single residues (p.Pro34del or p.Ser30del) in the ectodomain of BCMA, generate peptide variants that, although maintaining detectable surface BCMA expression, inhibit the binding of symmetrical monovalent anti-BCMA TCEs and hence abrogate their cytolytic activity. These events represent hotspot mutations in the BCMA extracellular domain with p.Pro34del and p.Ser30del detected in three and two out of six patients, respectively.

Therapy resistance due to these *TNFRSF17* mutations is not an all-or-none phenomenon; even though surface expression of p.Arg27Pro BCMA, for instance, was detectable with polyclonal anti-BCMA antibodies, monovalent anti-BCMA TCE binding was entirely abrogated by this missense mutation. These findings highlight that the commonly used anti-BCMA IHC antibodies, recognizing the cytoplasmic domain of BCMA or polyclonal flow cytometry antibodies that fail to detect pertinent extracellular domain mutations, are insufficient screening tools to accurately estimate the prevalence of BCMA mutational events leading to anti-BCMA CAR T/TCE resistance. Notably, p.Arg27Pro mutant BCMA did not universally negate the efficacy of all anti-BCMA therapies. An in-house CAR T with Ide-cel derived single-chain variable fragment (scFV)^21^ and an asymmetrical, BCMA-bivalent, bispecific antibody retained their cytolytic activity. Therefore, recognition of BCMA mutants by diagnostic or therapeutic anti-BCMA TCE is dependent not only on their targeted epitope specificities, but also on the structural design of these therapeutic molecules. Importantly, our findings also indicate that an acquired resistance to one anti-BCMA TCE may not necessarily translate into resistance to other anti-BCMA TCEs with different anti-BCMA Fab molecules, valency or structural TCE design.

Notably, BCMA extracellular domain mutations identified post-TCE did not impair the binding of its canonical ligand APRIL nor did it affect NF-κB downstream signaling. Of note, both patients with biallelic *TNFRSF17* deletions had clonal NF-κB pathway-activating mutations (*TRAF3* and *CYLD*). Whether pre-existing NF-κB-activating mutations predispose patients to biallelic *TNFRSF17* loss remains to be determined.

Last, we did not detect BCMA extracellular domain mutant subclones in the pre-therapy samples of the index patients. This highlights the dynamic nature of clonal antigen escape and underscores the importance of serial tumor sampling and genomic analysis to enhance the sensitivity of detecting emerging antigen escape clones and adjusting therapies accordingly.

With respect to GPRC5D, in the present study we have described cases of GPRC5D-negative relapse post-TCE with biallelic deletions or single CN loss coupled with multiple *GPRC5D* mutational events (single-nucleotide mutation, large frame deletion and balanced translocation). This convergent evolution under immune-therapeutic pressures indicates that this orphan gene can be more readily lost than *TNFRSF17* in MM cells.

The discovery of mutations in the BCMA ectodomain and *GPRC5D* that differentially attenuate CAR T/TCE binding to their cognate epitope, precipitating MM disease relapse, highlights the critical relevance of screening for these variants. Detailed characterization of these binding interactions will permit the rational design of next-generation T cell-redirecting agents and inform the optimal sequencing and combination of these immune-therapeutic approaches.

## Methods

### Patient CD138^+^ sample collection

All work with human samples was approved by the conjoint health research ethics board at the University of Calgary (HREBA.CC-21-0248), the health research ethics board at the University Hospital of Würzburg (Würzburg EK 8/21) and the City of Hope TGen institute health research ethics board (Western Institutional Review Board, protocol no. 20160566), and are consistent with the Declaration of Helsinki. Patient samples were obtained after obtaining written informed consent. Serial bone marrow aspirates were collected from patients treated with anti-BCMA CAR T/TCE (bb2121: protocol no. BB2121-MM-003, ClinicalTrials.gov identifier NCT03651128; teclistamab: protocol no. 64007957MMY1001, ClinicalTrials.gov identifier NCT03145181; elranatamab: protocol no. C1071001, ClinicalTrials.gov identifier NCT03269136 and protocol no. C1071005, ClinicalTrials.gov identifier NCT05020236) or anti-GPRC5D TCE (talquetamab: protocol no. 64407564MMY1002, ClinicalTrials.gov identifier NCT04108195) or other non-anti-BCMA/GPRC5D CAR T/TCE salvage treatments (anti-BCMA/GPRC5D naive) before therapy initiation and/or at the time of relapse. Overall the study cohort included 40 patients with MM treated with anti-BCMA (*n* = 21), anti-GPRC5D (*n* = 6), both anti-BCMA and anti-GPRC5D (*n* = 3) and a non-anti-BCMA/-GPRC5D regimen (*n* = 10) (Supplementary Table 1). The patients who were anti-BCMA/-GPRC5D naive (*n* = 10) were included in this analysis to enrich the cohort of samples used to determine the baseline frequency of *TNFRSF17* and *GPRC5D* mutagenic events in patients with MM.

CD138^+^ MM cells were isolated from bone marrow aspirates by Ficoll gradient separation of the mononuclear cell fraction followed by CD138^+^ magnetic bead incubation (Miltenyi Biotec, catalog no. 130-051-301) and column sorting. Isolated CD138^+^ cells were resuspended in 90% fetal bovine serum (FBS) with 10% dimethyl sulfoxide and stored at −80 °C until they were ready to be thawed and used. Samples were subjected to scRNA-seq, scCNV-seq analysis and/or bulk WGS as summarized in Supplementary Table 1. Samples from the University of Calgary were selected according to material availability across all patients treated with anti-BCMA, GPRC5D TCE or anti-BCMA CAR T. For the University Hospital Center Würzburg, samples were collected from patients who rapidly achieved a deep clinical response and subsequently rapidly progressed. The sample from City of Hope was selected based on progressive disease post-TCE therapy as part of a local personalized medicine study. For this sample, CD138^+^ MM cells were isolated on an Applied Cells MARS CS instrument after labeling with CD138-PE (phycoerythrin) and anti-PE magnetic beads. Clinical characteristics of analyzed patients are summarized in Supplementary Table 1. Study participants’ sex was determined based on their biological attributes. The present study and the consenting form did not include gender information and, therefore, gender information was not collected. Sex of the patients included in this analysis is listed in Supplementary Table 1.

### MLL WGS dataset

The WGS data were generated as a part of the 5,000 Genomes project, which was launched at the MLL Munich Leukemia Laboratory in 2017 with the aim of sequencing the genome and transcriptome of 5,000 patients with hematological malignancies to gain a more in-depth knowledge of their molecular profiles and genetic complexity. The evaluated WGS dataset of 4,995 patients includes patients with various myeloid and lymphoid neoplasms such as chronic myelogenous leukemia, myelodysplastic syndrome, chronic myelomonocytic leukemia, myeloproliferative neoplasms, mastocytosis, acute myeloid leukemia, mixed phenotype acute leukemia, B and T cell acute lymphoblastic leukemia, chronic lymphocytic leukemia, mantle cell lymphoma, follicular lymphoma, marginal zone lymphoma, lymphoplasmacytic lymphoma, high-grade B cell lymphoma, hairy cell leukemia, blastic plasmacytoid dendritic cell neoplasm, monoclonal gammopathy of undetermined significance, MM and mature T cell and natural killer cell neoplasms. Samples (bone marrow and/or peripheral blood) were collected by the MLL study group between 2005 and 2022. Genomes with a median coverage of 100× (151 bp paired-end) were sequenced on HiSeqX and NovaSeq 6000 instruments (Illumina).

### ScRNA-seq and scCNV-seq and analysis

Unbiased scRNA-seq and single-cell DNA profiling (scCNV-seq) of bone marrow-sorted CD138^+^ MM cells were conducted using the GemCode system (10× Genomics) according to the manufacturer’s protocols. In detail, for single-cell library preparation for RNA-seq, primary MM cells were processed according to 10× Genomics reagent Kits User Guide (CG00052 v.2 Chemistry). Cells were partitioned into nanoliter-scale gel bead in emulsions (GEMs) using 10× GemCode Technology. Primers containing (1) an Illumina R1 sequence, (2) a 16-bp 10× barcode, (3) a 10-bp unique molecular identifier (UMI) and (4) a poly(dT) primer sequence were incubated with partitioned cells, resulting in barcoded, full-length complementary DNA from poly(adenylated) messenger RNA. Silane magnetic beads were used to remove leftover biochemical reagents/primers, then complementary DNA was amplified by PCR. Enzymatic fragmentation and size selection were used to optimize cDNA amplicon size before library construction. R1 (read 1 primer sequence) was added during GEM incubation, whereas P5, P7, a sample index (i7) and R2 (read 2 primer sequence) were added during library construction via end repair, A-tailing, adapter ligation and PCR.

For single-cell DNA library generation for CNVs, single-cell suspensions of primary MM cells were processed according to 10× Genomics Reagent Kits User Guide (CG000153). Single cells were partitioned in a hydrogel matrix by combining with a CB polymer to form cell beads (CBs) using a microfluidic chip. Post-primary encapsulation, CBs were treated to lyse the encapsulated cells and denature the genomic DNA (gDNA). The denatured gDNAs in the CBs were then accessible to amplification and barcoding. A second microfluidic encapsulation step was then required to partition the CBs with 10× barcode gel beads to generate GEMs. Immediately after barcoding and amplification, 10× barcoded fragments were pooled and attached to standard Illumina adapters.

For all the single-cell methods, quality control and quantification were performed using a KAPA Library Quantification qPCR kit (Kapa Biosystems) on a BioRad quantitative (q)PCR instrument before preparing a single pool containing equimolar amounts of each library. This pool was then subjected to on-board cluster formation and sequencing on an Illumina NextSeq 500 sequencer with a high-output v.2.5 150-sequencing kit for RNA-seq and 300 cycle-sequencing kit for CNV-seq as per the standard Illumina protocols. After sequencing, bcl data were converted to fastq data files using the Illumina BCL2FASTQ utility.

Genomic sequence reads were treated with the CellRanger suite (cellranger v.3.1.0. and cellranger-dna v.1.1.0 for scRNA-seq and scCNV-seq, respectively) against the human reference genome GRCh38 with default parameters.

For gene expression data analysis, filtered feature barcode hierarchical data format 5 (HDF5) matrices created with a cellranger pipeline were imported into the R package Seurat^26^ (v.4) for normalization, scaling, integration, multi-modal reference mapping, clustering, dimensionality reduction, differential expression analysis and visualization. Cells with barcodes with <1,000 UMIs, >25,000 UMIs or >25% of mitochondrial reads were discarded. Plasma cells were identified based on expression of *TNFRSF17*, *CD38* or *SDC1* and *IGKC/IGLC* genes (UMI > 100), as well as negative expression of *CD3D/G/E*, *CD14* and *FCGR3A*.

For CNV data analysis, HDF5 matrices (cnv_data.h5) and CN files (node_unmerged_cnv_calls.bed) generated by the cellranger-dna suite v.1.1.0, as well as heatmap CN data-generated Loupe scDNA (v.1.1.0), were processed with customized R scripts (available in the Github repository: https://github.com/nbahlis/Myeloma_Immunotherapy_Antigen_Escape). For readability purposes and CN estimates, more than four copies were reduced to four and marked ‘≥4’.

### WGS and analysis

WGS was performed at different sites based on the location of patient sample acquisition.

For samples collected at the University of Calgary, WGS was performed at the New York Genome Center (NYGC) and libraries were prepared using the Truseq DNA Nano Library Preparation Kit (Illumina 20015965) in accordance with the manufacturer’s instructions. Briefly, 100 ng of DNA was sheared using a Covaris LE220 sonicator (adaptive focused acoustics). DNA fragments underwent bead-based size selection and were subsequently end-repaired, adenylated, ligated to Illumina sequencing adapters and amplified. Final libraries were quantified using the Qubit Fluorometer (Life Technologies) or Spectramax M2 (Molecular Devices) and Fragment Analyzer (Advanced Analytical) or Agilent 2100 BioAnalyzer. Libraries were sequenced on an Illumina Novaseq 6000 sequencer using 2× 150-bp cycles. Post-sequencing analysis and somatic variants, calls were performed using the NYGC pipeline. Briefly, SNVs were called integrating Mutect2 (v.4.1), Strelka (v.2.4.7) and Lancet (v.1.1.0); indels (insertions and deletions) were called using Mutect2, Manta (v.0.28.0), Strelka2 (v.2.9.10) and Lancet, SvABA (v.1.2.0); and structural variants (SVs) were called using Manta, SvABA and Lumpy (v.0.3.1). Finally CN changes were characterized using ASCAT (v.3.1.2)^27^. Details of the NYGC somatic variant analysis can be found at this link: chrome-extension://efaidnbmnnnibpcajpcglclefindmkaj/https://www.nygenome.org/bioinformatics/wp-content/uploads/2019/06/SomaticPipeline_v6.0_Human_WGS.pdf.

CNV analysis and purity were called using ASCAT (https://github.com/VanLoo-lab/ascat) and Battenberg (https://github.com/Wedge-lab/battenberg). The Dirichlet process was used to reconstruct the phylogenetic tree of patients with multiple samples collected at different timepoints^28^.

For patient samples collected at the University Hospital Center Würzburg, WGS libraries were prepared from 1 µg of DNA from CD138^+^ purified cells with the TruSeq PCR-free library prep kit and 2× 151-bp paired-end sequences were generated on a NovaSeq 6000 instrument (Illumina) with a median coverage of 84× (2020), 149× (2022) and 43× (normal). A tumor/matched normal workflow was used for variant calling. Reads were aligned to the human reference genome (GRCh37) using Isaac aligner (v.03.16.02.19) through the BaseSpace WGS app v.5 (Illumina) with default parameters. SNVs were called with Strelka Somatic Variant Caller (v.2.4.7) and SVs with Manta (v.0.28.0). CNVs were called using GATK4 (v.4.0.8.1, Broad Institute). Each variant with a PASS flag was queried against the gnomAD database (v.2.1.1) and variants with global population frequencies >0.05% were excluded to reduce germline calls. Further analysis was performed on protein-altering and splice-site variants only.

For the patient sample collected at City of Hope, PCR-free WGS libraries were constructed at TGen on an Agilent Bravo liquid handler from 100 ng of DNA using Watchmaker DNA Library Prep with Fragmentation with a final 0.5× post-ligation clean-up with SPRI beads. After qPCR quantification, diluted libraries were sequenced on a NovaSeq 6000 sequencer with 2× 151-bp reads. Somatic data analysis was performed using the Phoenix v.1.2.0 workflow (https://github.com/tgen/phoenix) on samples with >100× tumor and >40× normal coverage.

### Patient CD138^+^ MM cell BCMA and GPRC5D expression detection by flow cytometry

Frozen CD138^+^ samples from the tissue bank were thawed and washed twice with RPMI medium. Samples were resuspended in 100 µl of cell-staining buffer (CSB; BioLegend, catalog no. 420201) and incubated with anti-BCMA or anti-GPRC5D antibodies at 4 °C for 30 min. All flow cytometry antibodies used in the present study are listed in Supplementary Table 7a. Stained cells were washed and resuspended in 500 µl of CSB for flow cytometry analysis; 6,000–10,000 live events (based on forward and side-scatter plots) were recorded where possible, barring limited primary MM cell availability from biopsy samples. All flow cytometry experiments were conducted using the Beckman CytoFLEX flow cytometer. BCMA surface protein expression levels were determined based on median fluorescent intensities (MFIs) on single parameter histograms. All flow cytometry experiment analysis and figures in the present study were made using Kaluza Analysis Software 2.1 (Beckman Coulter). The gating strategy is shown in Supplementary Fig. 13.

### Digital PCR validation

Mutations were verified using the QuantStudio 3D Digital PCR system (Thermo Fisher Scientific). Genotyping assays were designed using the customized TaqMan Assay Design Tool with wild-type genotyping probes VIC labeled and mutant probes FAM labeled. Primers and probes sequences are listed in Supplementary Table 7c. Reactions consisted of 10 ng of DNA, 7.5 μl of dPCR Master Mix v.2 and 0.75 μl of 20× genotyping assay in a total volume of 15 μl. The dPCR reactions were loaded on to a QuantStudio 3D Chip v.2, then sealed and placed on a ProFlex Flat PCR thermocycler. The following thermocycler conditions were used: 96 °C, 10 min (60 °C, 2 min and 98 °C, 30 s) ×39 cycles, 60 °C, 2 min. Chips were then read using the QuantStudio 3D dPCR Instrument. The dPCR analysis was performed using QuantStudio 3D AnalysisSuite Software v.3.1.6.

### BCMA cloning and BCMA K562 cell-line generation

Human *TNFRSF17* cDNA sequence was taken from the CCDS sequence data available from the National Center for Biotechnology Information (NCBI) CCDS database (CCDS ID: CCDS10552.1)^29^. Arginine at amino acid position 27 (wild-type) was mutated to proline to generate p.Arg27Pro mutant BCMA by exchanging the codon CGA to CCA. The wild-type and p.Arg27Pro mutant *TNFRSF17* sequences were cloned into the pLX307 lentiviral plasmid backbone (Addgene, catalog no. 17734) under EF-1α promoter and tagged with the V5 sequence (GKPIPNPLLGLDST) at the carboxy terminus of *TNFRSF17*.

Template DNA and primers were ordered from Integrated DNA Technologies (IDT) as a customized DNA oligo. The KAPA HiFi HotStart ReadyMix PCR kit (Kapa Biosystems, catalog no. KM2605) and QIAquick Gel Extraction and PCR & Gel Cleanup kit (QIAGEN, catalog no. 28704) were used for DNA amplification and purification. Restriction enzymes NheI (New England Biolabs (NEB), catalog no. R3131M) and EcoRV (NEB, catalog no. R0195S) were used for digestion. Ligation was performed using overnight T4 DNA ligase (NEB, catalog no. M0202L) incubation.

For the p.Pro33Ser, p.Ser30del and p.Pro34del BCMA variants, site-directed mutagenesis was performed on the wild-type TNFRSF17 pLX307 plasmid generated above. Three sets of forward and reverse primers were designed and ordered from IDT to induce missense mutation of Pro33 to serine (CCT to TCT) or in-frame deletion of amino acid residue Ser30 or Pro34. KAPA HiFi HotStart ReadyMix PCR kit (Kapa Biosystems, catalog no. KM2605) was used for the site-directed mutagenesis.

Transformation was carried out using NEB stable competent *Escherichia coli* cells (NEB, catalog no. C3040H) with 42 °C heat shock. Bacterial colonies selected from the ampicillin agar plate were processed using QIAprep Spin Miniprep Kit (QIAGEN, catalog no. 27106). All plasmid DNA sequences were validated by Sanger sequencing to verify wild-type and mutant BCMAs. EndoFree Plasmid Maxi Kit (QIAGEN, catalog no. 12362) was used to generate bulk transfer plasmids. A second-generation lentiviral packaging system was used to assemble the transfer plasmid with the psPAX2 packaging plasmid (Addgene, catalog no. 12260) and pMD2.G envelope plasmid (Addgene, catalog no. 12259) in HEK293T cells using a calcium phosphate transfection kit (Thermo Fisher Scientific, catalog no. K278001). HEK293T cells were cultured in Dulbecco’s modified Eagle’s medium (Gibco, catalog no. 11965-092) with 10% FBS and 1% penicillin–streptomycin. After 48 h of incubation, the supernatant was collected and filtered using a 0.45-μm filter, followed by ultracentrifugation at 90,000*g*. The lentiviral pellet was resuspended in phosphate-buffered saline (PBS) and stored in −80 °C until further use.

K562 cells were resuspended in RPMI medium at a density of 750,000 cells ml^−1^ and transduced with BCMA lentivirus using polybrene (10 µg ml^−1^). The BCMA expression level was assessed using anti-BCMA antibodies (Supplementary Table 7a) by flow cytometry at 48 h after transduction and weekly thereafter. The K562 cell lines were cultured in full RPMI medium (Gibco, catalog no. 11875-093) (containing 10% FBS, 1% penicillin–streptomycin and 0.2% normocin) at 37 °C and 5% CO_2._

The sources of all reagents, buffers, oligonucleotide sequences and cell lines used in the present study are listed in Supplementary Table 7.

### BCMA CAR T generation

Anti-BCMA CAR design has been previously described^21^. The cloning of the CAR sequence (light chain scFv–heavy chain scFv–CD8 transmembrane domain–41BB–CD3ζ) into the pLX307 lentiviral plasmid backbone, as well as the generation of anti-BCMA CAR lentivirus, were performed using the same cloning and lentivirus purification protocols as the BCMA cloning procedure outlined above.

Healthy donor PBMCs were collected from Ficoll gradient centrifugation and CD3^+^ T cells were sorted by incubating with CD3^+^ beads (Miltenyi Biotec, catalog no. 130-050-101) followed by magnetic column separation. CD3^+^ T cells were resuspended at 1 × 10^6^ ml^−1^ in ImmunoCult-XF T cell Expansion Medium (Stemcell, catalog no. 10981) with IL-2 (10 ng ml^−1^) and activated using ImmunoCult Human CD3/28T cell activator (Stemcell, catalog no. 10971) at 25 µl ml^−1^ concentration per the manufacturer’s protocol. Then, 24 h after activation, the T cells were transduced with anti-BCMA CAR lentivirus with polybrene followed by centrifugation at 180*g* for 1 min. Transduction efficiency was verified at 48 h by checking PE-labeled BCMA peptide (AcroBiosystem, catalog no. BCMA BCA-HP2H2) binding on flow cytometry analysis.

### TCE-binding assay

Anti-BCMA TCEs, teclistamab, elranatamab and alnuctamab, used for the TCE-binding assays, were obtained from Janssen Pharmaceuticals, Pfizer and Bristol Myers Squibb, respectively. Parental or BCMA-expressing K562 cell lines were resuspended in RPMI medium (1 × 10^6^ cells ml^−1^) and incubated with 10 nM TCE for 30 min at room temperature. The cells were then resuspended in CSB and incubated with AF-488 anti-IgG4 Fc, PE anti-IgG2 Fc or APC (allophycocyanin)-anti-IgG Fc flow antibodies (Supplementary Table 7a) for an additional 15 min at room temperature. Cells were washed twice with CSB before flow cytometry analysis; 10,000 live events (based on forward and side-scatter plots) were recorded and anti-IgG antibody expression levels were determined based on MFIs on single parameter histograms.

### APRIL-binding assay

Fc-tagged APRIL trimer (ACRO Biosystems) was used to assess APRIL binding in K562 cell lines lentivirally transduced to stably express wild-type or mutant BCMAs. Cells were resuspended in CSB and incubated with APRIL at the indicated concentrations for 30 min at room temperature, washed once with PBS and incubated with APC-anti-IgG Fc flow antibody for an additional 15 min at room temperature. Cells were washed and analyzed by flow cytometry.

### TCE and CAR T cytotoxicity assay

Healthy donor PBMCs were collected from Ficoll gradient centrifugation and washed with PBS for use as effector cells in co-culture assays. Parental or BCMA-expressing K562 cell lines were stained with CellTrace Violet (CTV; Thermo Fisher Scientific, catalog no. C34557) for 20 min at 37 °C protected from light. PBMCs were co-cultured with CTV-stained K562 cell lines in full RPMI medium at a 10:1 effector:target ratio with or without bispecific antibodies (teclistamab (Janssen), elranatamab (Pfizer), alnuctamab (Bristol Myers Squibb) or anti-BCMA BiTE (BPS Bioscience, catalog no. 100689)), tested at varying concentrations (0.01–100 nM). The co-cultures were incubated for 24–48 h at 37 °C and 5% CO_2_. Calcein AM (Thermo Fisher Scientific, catalog no. C1430) and propidium iodide (PI; BioVision, catalog no. 1056) were used to stain the cells before flow cytometry as per the manufacturers’ protocols. Events, 4,000–10,000, of CTV-positive cells were collected per treatment condition. After gating on CTV-positive cells in the BV421 channel, the cells were displayed on a two-parameter dot (density) plot (PE/Texas Red in vertical axis for PI and FITC in horizontal axis for calcein AM) to determine the proportion of PI- versus calcein AM-positive cells. A cluster of cells staining strongly positive for calcein AM was considered viable and their gated percentage was used to compare target cell viabilities across different treatment conditions.

### Structural analysis of the wild-type and mutant BCMAs

BCMA three-dimensional structures were modeled using a template-based algorithm implemented on the GalaxyTBM tool (PMID: 22883815). Predicted structures were subsequently refined and used as the input models for molecular dynamics simulations carried out by the Gromacs package^30^. Interaction of BCMA wild-type and mutant-type structures with teclistamab and elranatamab was simulated with site-directed molecular docking studies via the ClusPro server^31^.

### Soluble BCMA ELISA assay

Parental or BCMA-expressing K562 cell lines were resuspended in full RPMI medium (1 × 10^6^ ml^−1^) with or without 6.2 nM of the γ-secretase inhibitor Nirogacestat PF-3084014 (MedChemExpress, catalog no. HY-15185) and cultured overnight at 37 °C and 5% CO_2_. The supernatant was collected for a soluble BCMA ELISA assay. Human BCMA/TNFRSF17 DuoSet ELISA kit (R&D Systems, catalog no. DY193) was used per the manufacturer’s protocol to compare the quantity of soluble BCMA produced by the cell lines. Optical densities were measured using a SpectraMax iD3 microplate reader set to 450 nm and 570 nm per protocol.

### BCMA protein stability assay

K562 cells were resuspended in full RPMI medium and treated with 50 µg of cycloheximide (10 µg ml^−1^) for 0, 4 or 18 h. Cells were centrifuged (180*g* for 5 min), washed with PBS and treated with radioimmunoprecipitation assay (RIPA) lysis buffer for 30 min on ice. Cell lysates were collected and protein quantification was performed using the DC protein assay (BioRad) with the optical density reading set at 750 nm using a SpectraMax iD3 microplate reader. Protein lysates were loaded and run on NuPAGE 4–12% gel and transferred to a nitrocellulose membrane. Antibodies used for BCMA detection by western blotting were: 1:4,000 V5-probe sv5-pk mouse (Santa Cruz, catalog no. sc-58052); 1:5,000 anti-mouse IgG horseradish peroxidase (HRP) linked (Cell Signaling, catalog no. 7076S); 1:4,000 glyceraldehyde 3-phosphate dehydrogenase (14C10) rabbit (Cell Signaling, catalog no. 2118L); and 1:5,000 anti-rabbit IgG HRP linked (Cell Signaling, catalog no. 7074S).

### Western blotting and ELISA for NF-κB activation assay

562 cells transduced to express wild-type or mutant BCMA were resuspended in serum-free RPMI medium for 48 h. Fc-tagged APRIL trimer (300 ng ml^−1^) was added at the indicated timepoints. For total cell lysate collection, cells were centrifuged, washed with PBS and treated with RIPA lysis buffer for 30 min on ice. Protein lysates were loaded and run on NuPAGE 4–12% gel and transferred to a nitrocellulose membrane. Membranes were blotted with antibodies for total ERK (1:2,000), phospho-pp44/42 MAPK (Erk 1/2) (Thr202/Tyr204) (1:2,000) and secondary staining with 1:3,000 anti-rabbit IgG HRP-linked antibody.

NF-κB p65 nuclear translocation was assessed in the K562 cells expressing wild-type or mutant BCMAs after stimulation with APRIL at the indicated time using NF-κB ELISA (TransAM NF-κB p65 kit, Active Motif, catalog no. 40096). Nuclear extraction was performed using the Nuclear Extraction kit (Active Motif, catalog no. 40010) per the manufacturer’s protocol. In brief, cells were collected and washed with PBS supplemented with phosphatase inhibitor. Cells were treated with hypotonic buffer on ice and centrifuged to remove the cytoplasmic fraction, and the resulting pellet was resuspended in complete lysis buffer to extract the nuclear fraction. Protein was quantified using the ProStain protein quantification kit (Active Motif, catalog no. 15001) and 10 µg of nuclear extract was loaded per well on the TransAM NFkB ELISA plate. Primary NF-κB antibody and secondary HRP-conjugated antibody were used at a 1:1,000 ratio for 1-h incubation each. Plates were incubated for 5 min after adding the developing solution and the optical density was read at 450 nm.

### Immunohistochemistry staining for GPRC5D expression on primary CD138^+^ samples

MM cell lines OPM2 (positive control) and KMS12PE (negative control) were pelleted, fixed and resuspended in approximately 30 × 10^6^ cells per 100 µl of Histogel (Thermo Fisher Scientific) and processed into paraffin-embedded blocks. Using the Leica Bond RX, 4-µm sections of the cell line blocks and biopsies of patients with MM were deparaffinized, antigen retrieved (pH 8.0, EDTA, 99 °C for 56 min) and single stained with CD138 (clone MI15, 1:500) and GPCR5D (clone 6D9, 1:50), separately as previously described^7^. The reaction was visualized using BOND Polymer Refine Detection system with 3,3′-diaminobenzidine tetrahydrochloride (DAB) followed by hematoxylin as counterstain.

### Statistical analysis

Experiments involving primary patient samples were conducted once, given the limited patient sample availability.

Data processing for cytotoxicity experiments and dose–response curve (DRC) generation were performed using GraphPad Prism v.9. For the DRC, TCE doses were log(transformed) and log(inhibitor) versus response curve was generated using nonlinear regression and asymmetrical 95% confidence intervals were calculated. For comparison of the K562 target cell viability in the TCE and CAR T cytotoxicity assays, Student’s *t*-test was performed without adjustments for multiple comparisons using the R function pairwise.t.test() to generate *P* values. The two-way analysis of variance (ANOVA) test was computed using the R function aov(). The Lollipop plots were generated using the trackviewer package lolliplot() function in R (v.4.2.2)^32^.

### Reporting summary

Further information on research design is available in the Nature Portfolio Reporting Summary linked to this article.

## Online content

Any methods, additional references, Nature Portfolio reporting summaries, source data, extended data, supplementary information, acknowledgements, peer review information; details of author contributions and competing interests; and statements of data and code availability are available at 10.1038/s41591-023-02491-5.

## Supplementary information

Supplementary Information Supplementary Figs. 1–13, and Table Legends, and References.

Reporting Summary

Supplementary Tables Supplementary Tables 1–8.

Supplementary Data 1 Source data for Supplementary Fig. 2d.

## Source data

Source Data Figs. 4e,f and Extended Data Fig. 4c Statistical source data for Fig. 4e,f, and Extended Data Fig. 4c.

Source Data Extended Data Figs. 4e and 7d Unprocessed blots for Extended Data Figs. 4e and 7d.

## Data Availability

ScCNV-seq and scRNA-seq datasets are available at the NCBI’s Gene Expression Omnibus (which automatically makes a Sequence Read Archive deposit) under the following accession no.: GSE226336. Genomic data of patients with MM enrolled within the CoMMpass trial (NCT01454297) were generated as part of the MMRF Personalized Medicine Initiative (https://research.themmrf.org). Source data are provided with this paper.

## Code availability

All analyses were performed using publicly available software as described in Methods. Raw scripts to generate scCNV-seq analysis figures included in this paper are available under the Github repository (https://github.com/nbahlis/Myeloma_Immunotherapy_Antigen_Escape).
