# Supplementary material for: Mechanisms of antigen escape from BCMA- or GPRC5D-targeted immunotherapies in multiple myeloma
Source: Nat Med. 2023 Aug 31;29(9):2295–306. doi: 10.1038/s41591-023-02491-5 (PMC10504087; doi:10.1038/s41591-023-02491-5)
Supplement: Supplementary file 1 — Supplementary Figs. 1–13, and Table Legends, and References. [file 41591_2023_2491_MOESM1_ESM.pdf]

nature medicine

Article

https://doi.org/10.1038/s41591-023-02491-5

Mechanisms of antigen escape from BCMA- or GPRC5D-targeted immunotherapies in multiple myeloma

In the format provided by the authors and unedited

Supplementary Figure 1

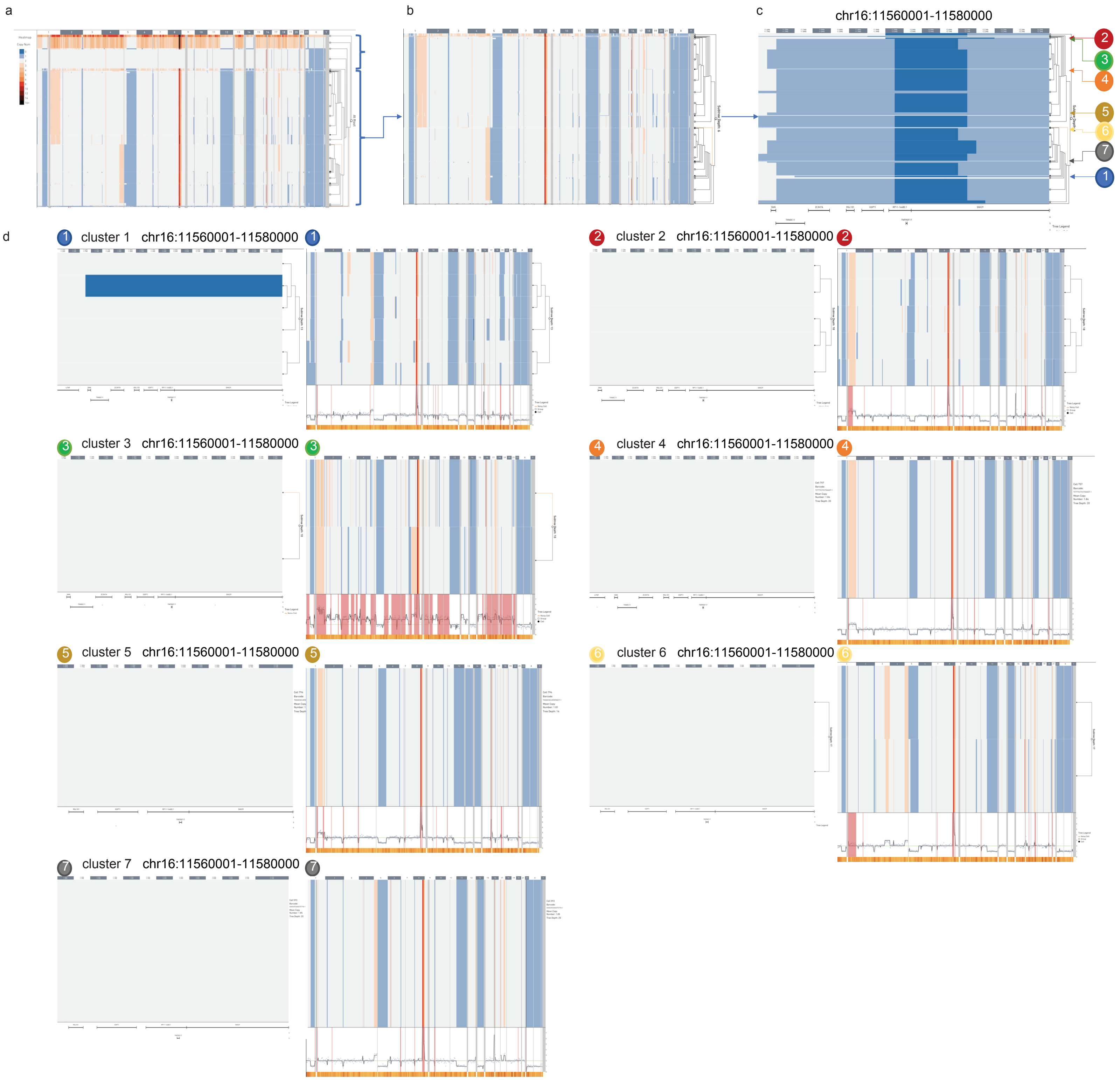

Supplementary Fig, 2

a

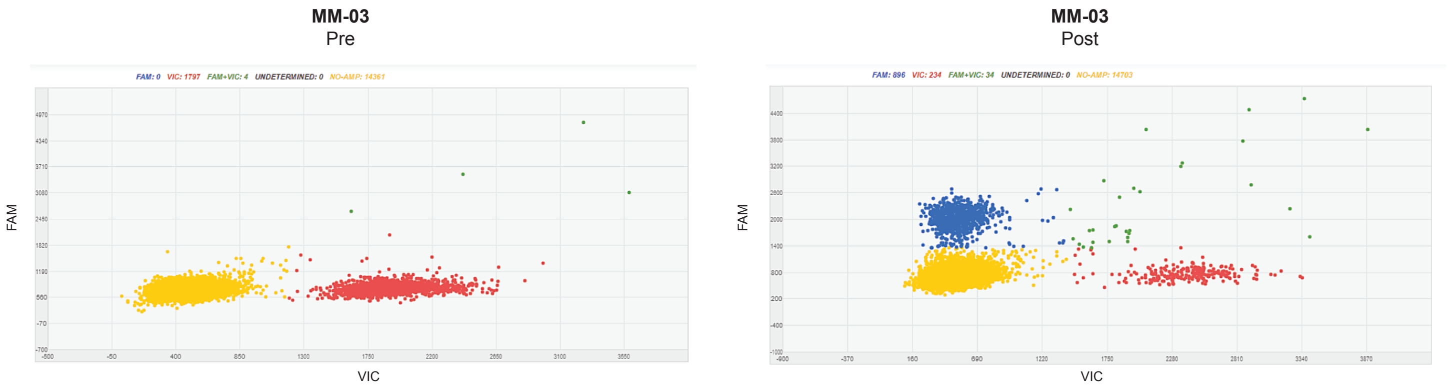

b

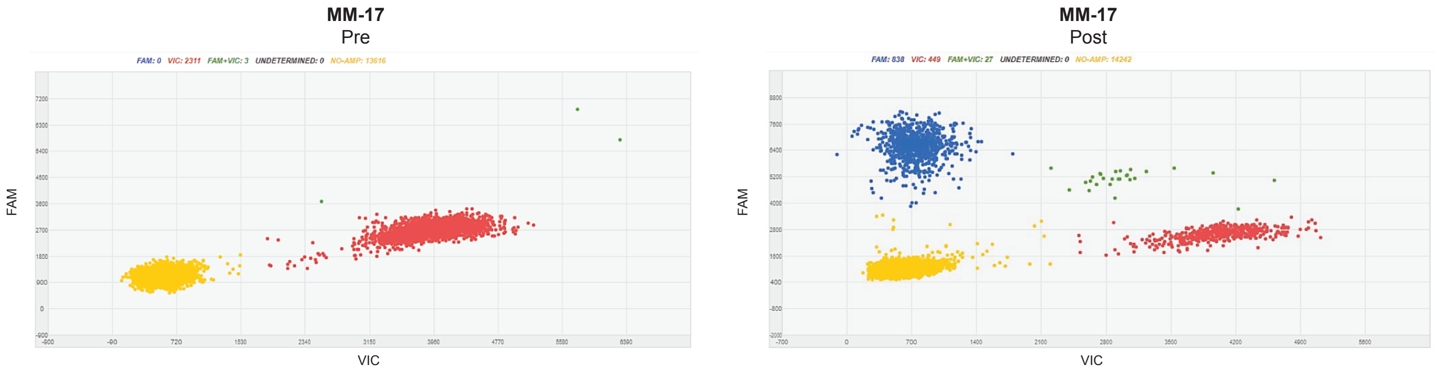

c

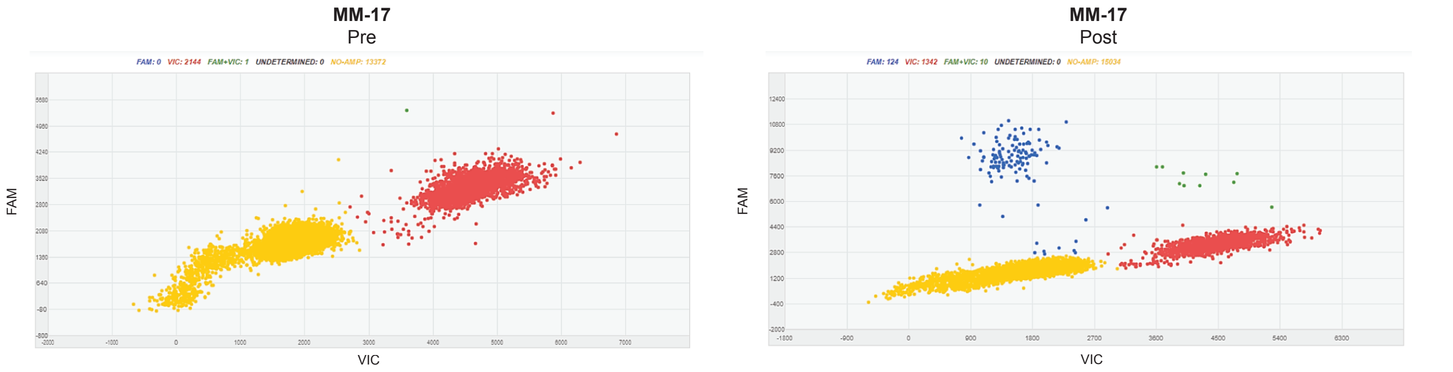

d

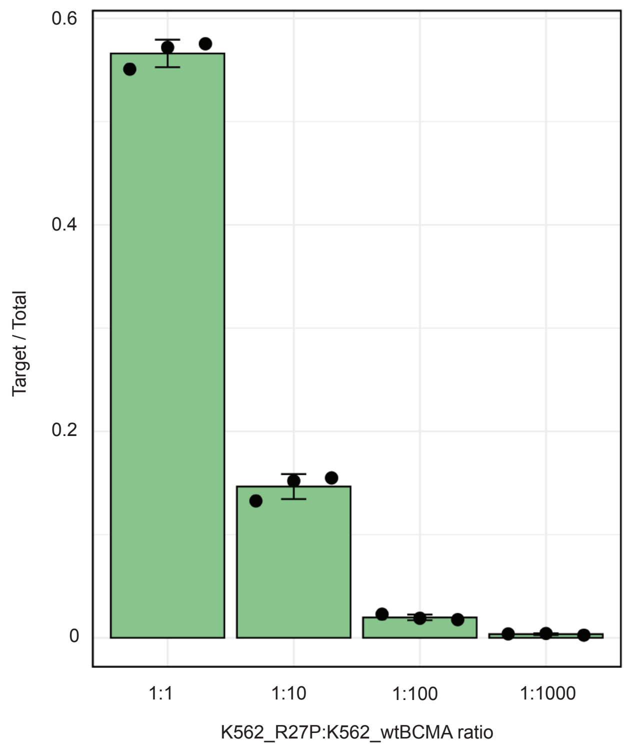

Supplementary Fig. 3

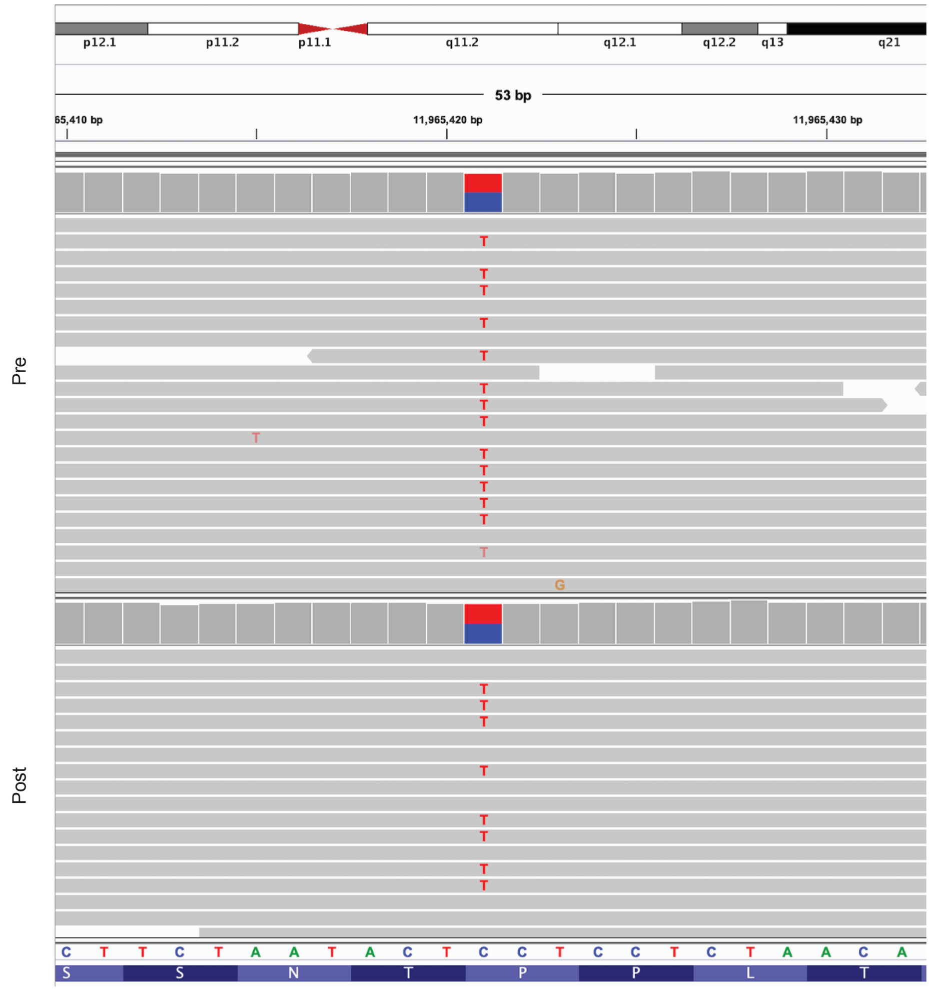

NPIPB2

Supplementary Fig. 4

a

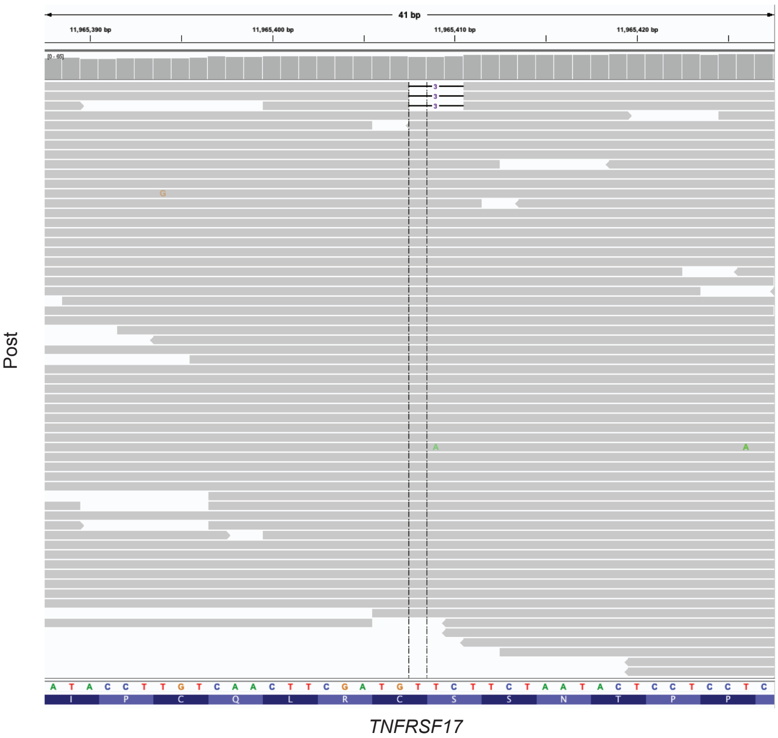

b

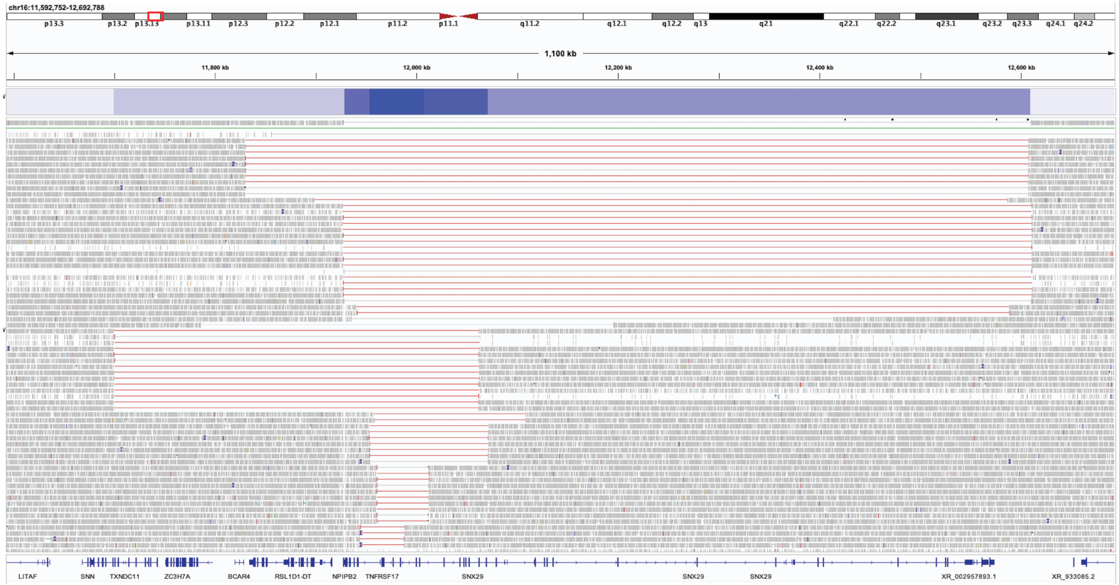

Supplementary Fig. 5

a

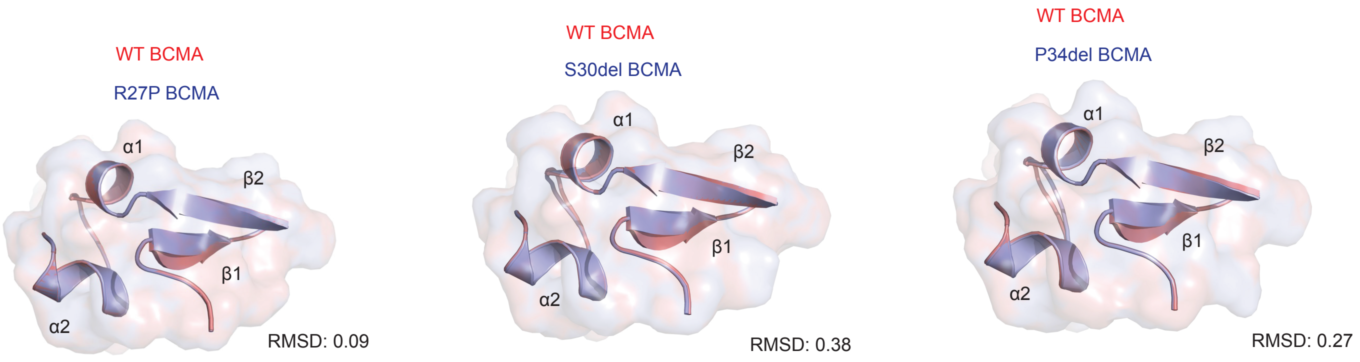

b

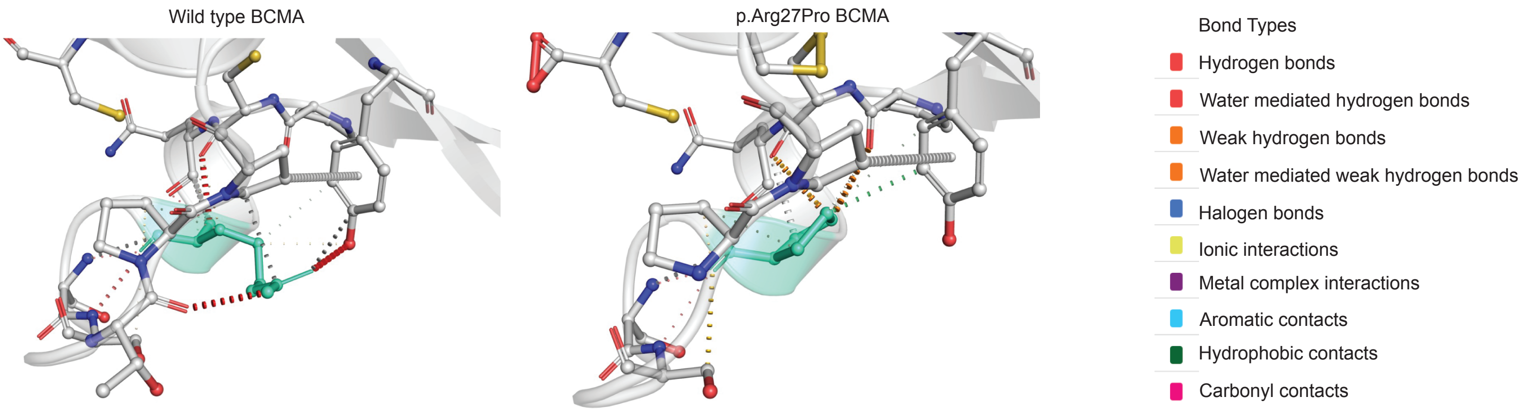

c

Δ Vibrational Entropy Energy Between Wild type and Mutant (p.Arg27Pro)
$\Delta\Delta S_{vib}$ ENCoM: -0.153 kcal.mol^{-1}.K^{-1} (Decrease of molecular flexibility)

Δ Vibrational Entropy Energy | Visual Representation

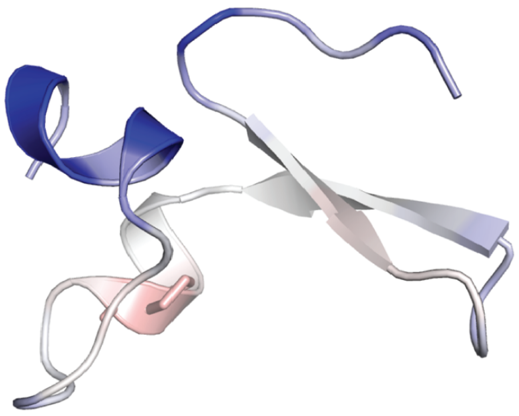

Prediction Outcome
ΔΔG: -0.426 kcal/mol (Destabilizing)

NMA Based Predictions
ΔΔG ENCoM: 0.123 kcal/mol (Destabilizing)

Other Structure-Based Predictions
ΔΔG mCSM: -0.906 kcal/mol (Destabilizing)
ΔΔG SDM: -1.530 kcal/mol (Destabilizing)
ΔΔG DUET: -1.155 kcal/mol (Destabilizing)

Amino acids colored according to the vibrational entropy change upon mutation.

- BLUE represents a rigidification of the structure
- RED represents a gain in flexibility

Supplementary Fig. 6

a

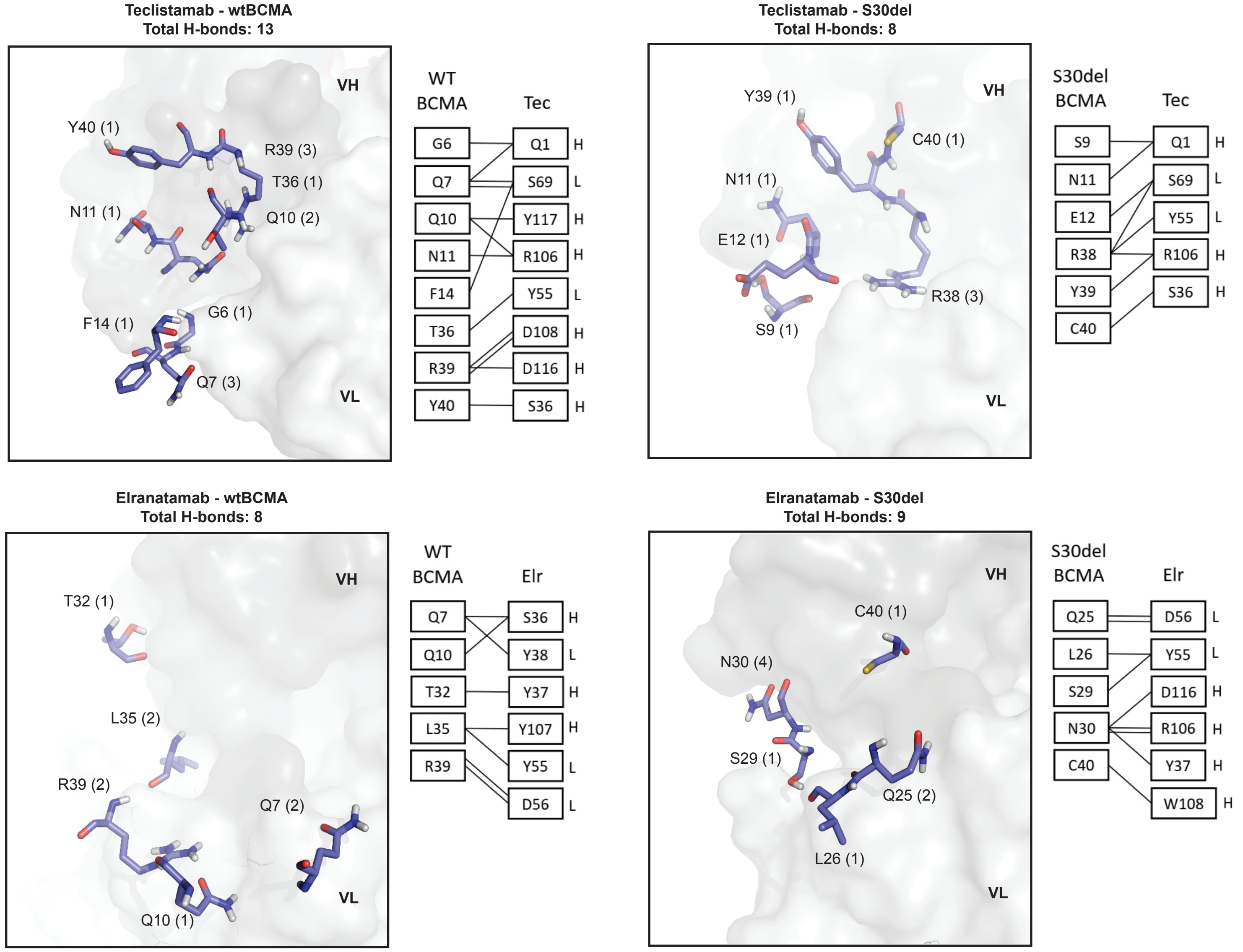

b

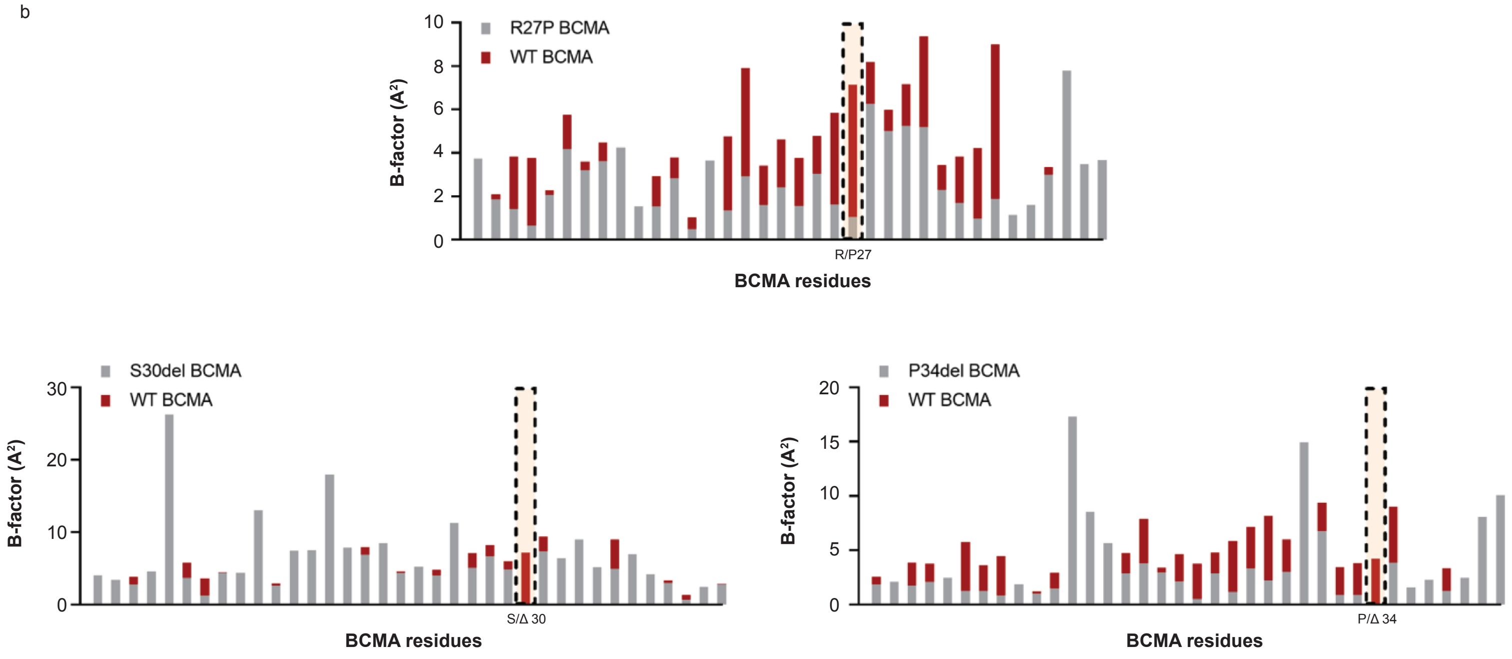

Supplementary Fig. 7

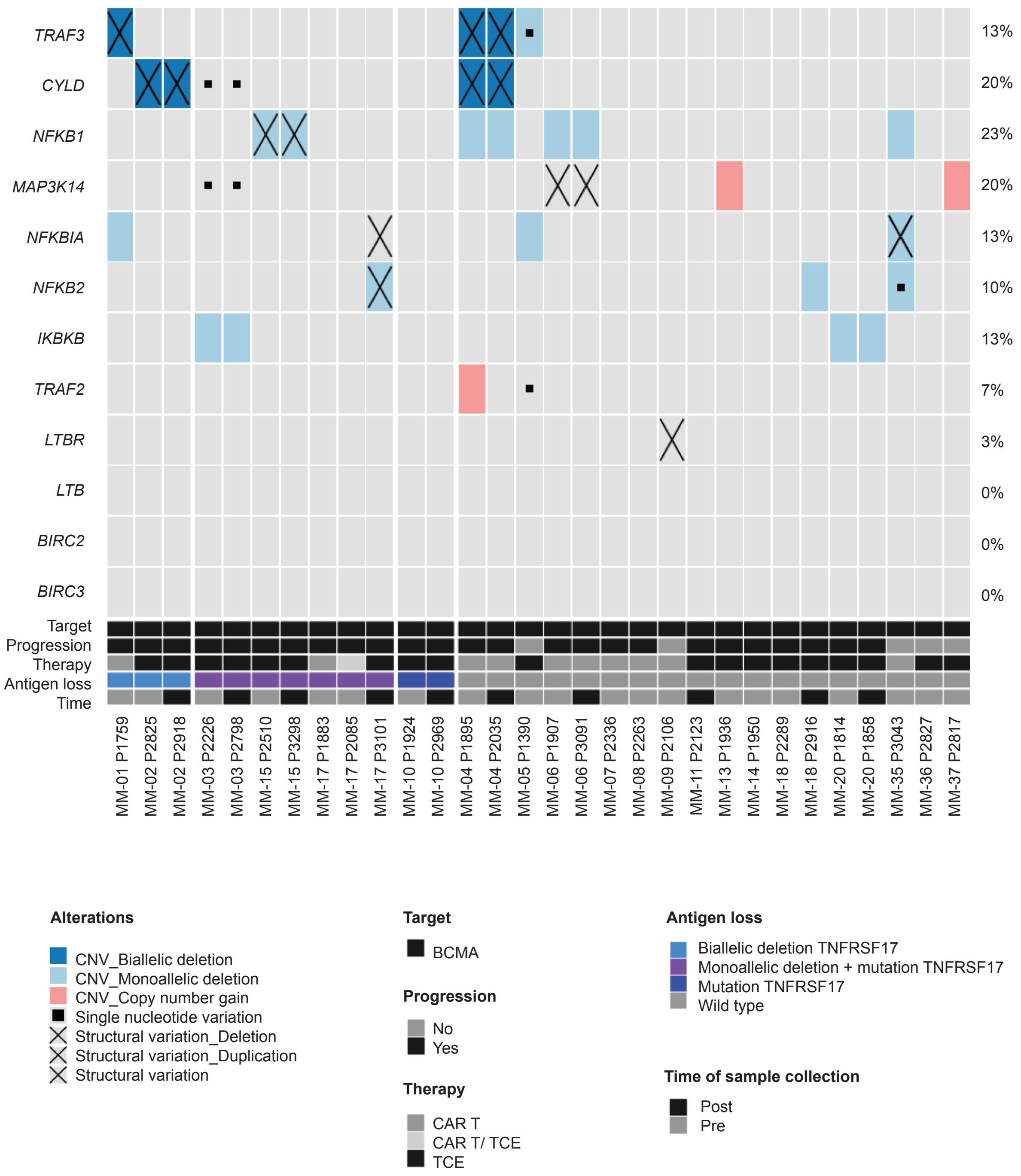

Supplementary Fig.8

a

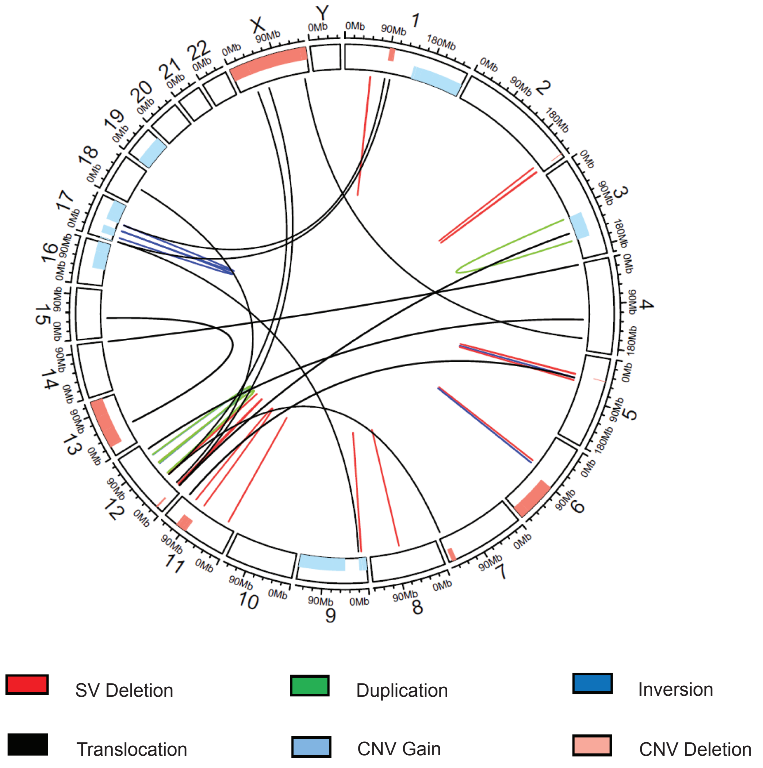

b

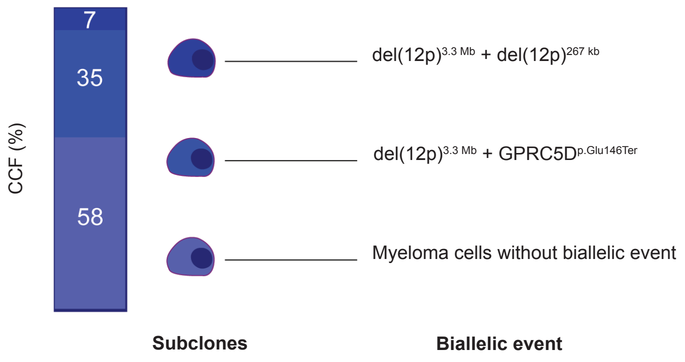

Supplementary Fig.9

a

MM-02
Pre

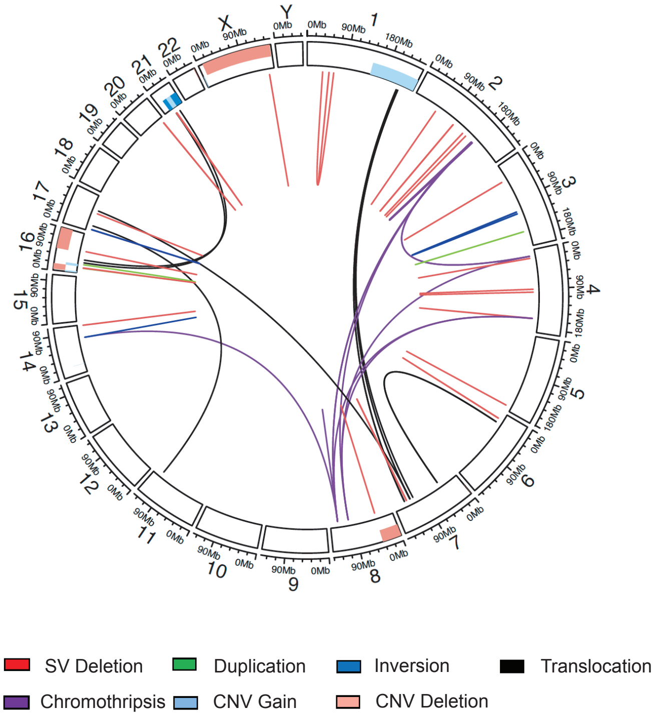

b

MM-02
Pre

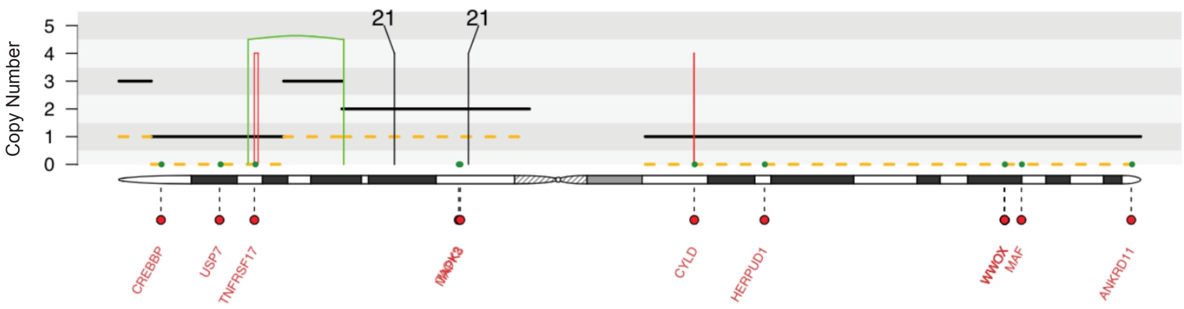

Supplementary Fig.10

MM-06

P1907 – chr12 – APOBEC = 0.0555845855428802 – Total Mutations 10358

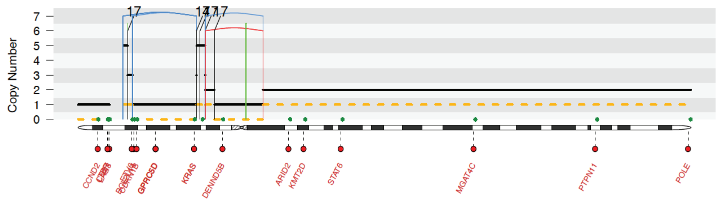

MM-08

P2263 – chr12 – APOBEC = 0 – Total Mutations 10805

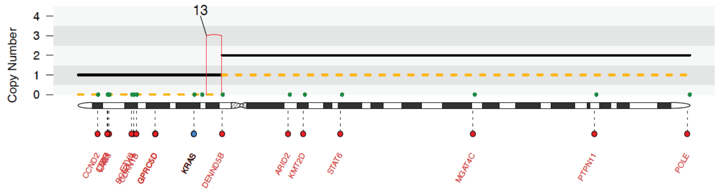

MM-18

P2289 – chr12 – APOBEC = 0.044489349390697 – Total Mutations 15884

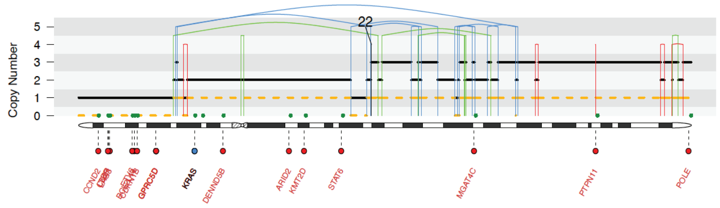

MM-20

P1814 – chr12 – APOBEC = 0.034841798481987 – Total Mutations 6461

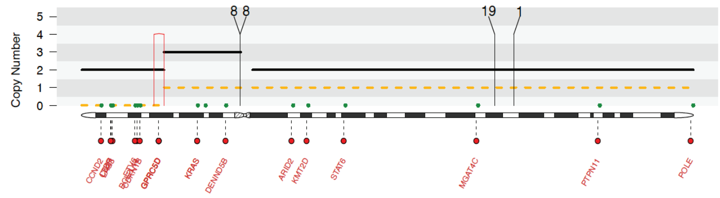

P2222 – chr12 – APOBEC = 0.0362062667915913 – Total Mutations 20813

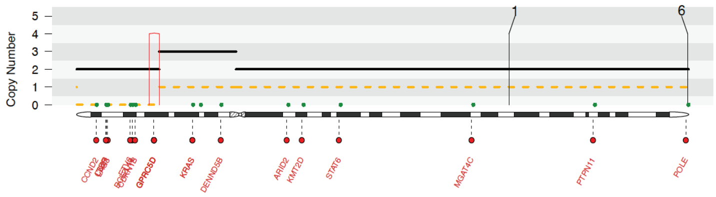

MM-21

P2337 – chr12 – APOBEC = 0.0506001072782965 – Total Mutations 14153

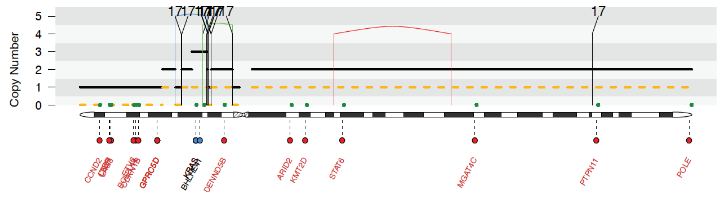

MM-36

P2827 – chr12 – APOBEC = 0.0326526029111443 – Total Mutations 6279

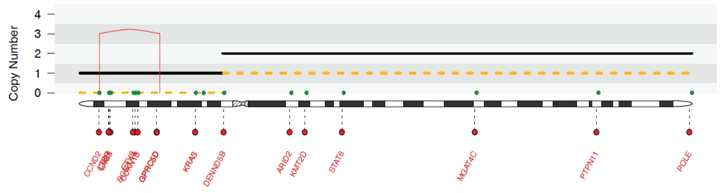

Supplementary Fig.11

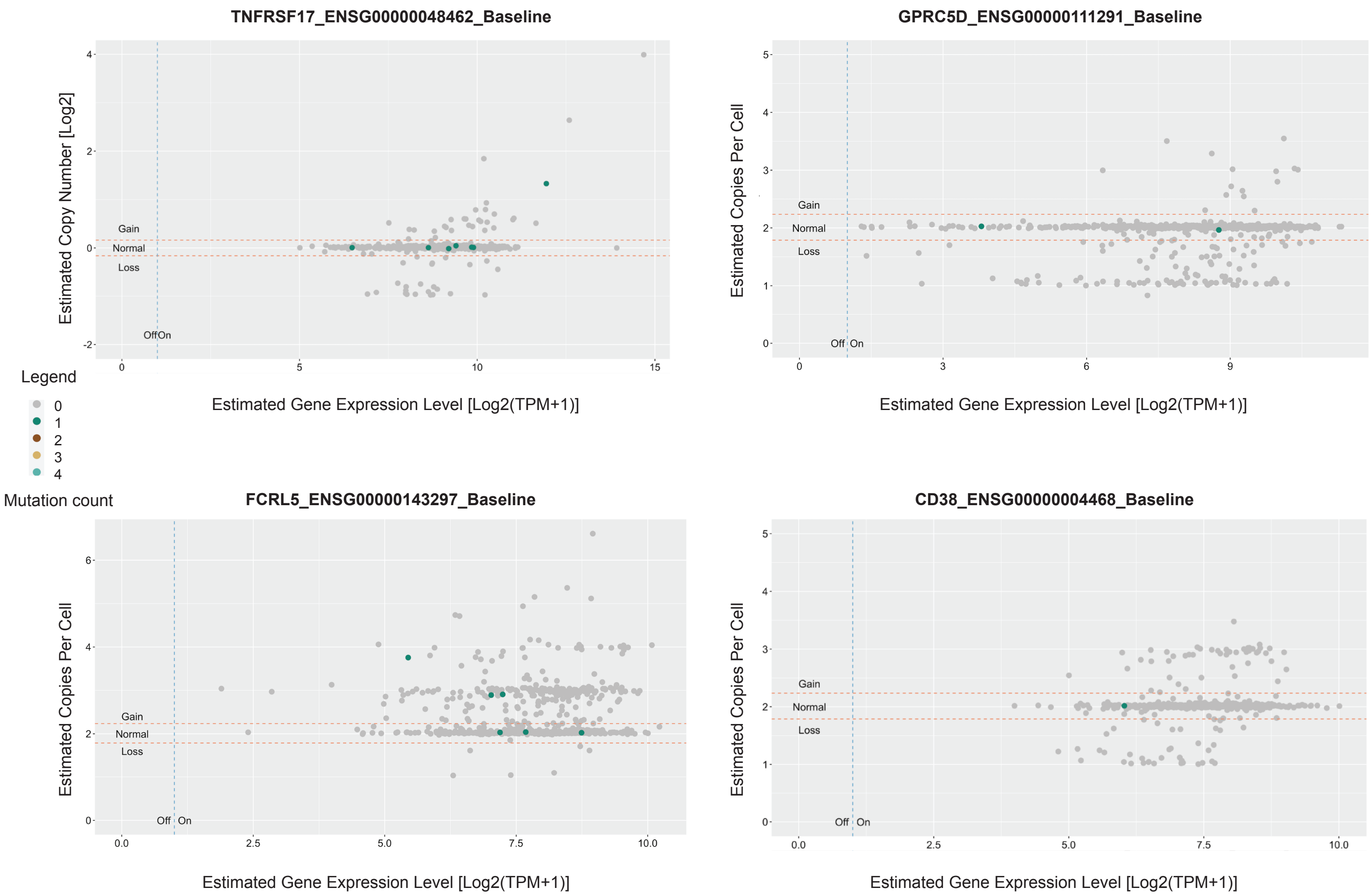

Supplementary Fig.12

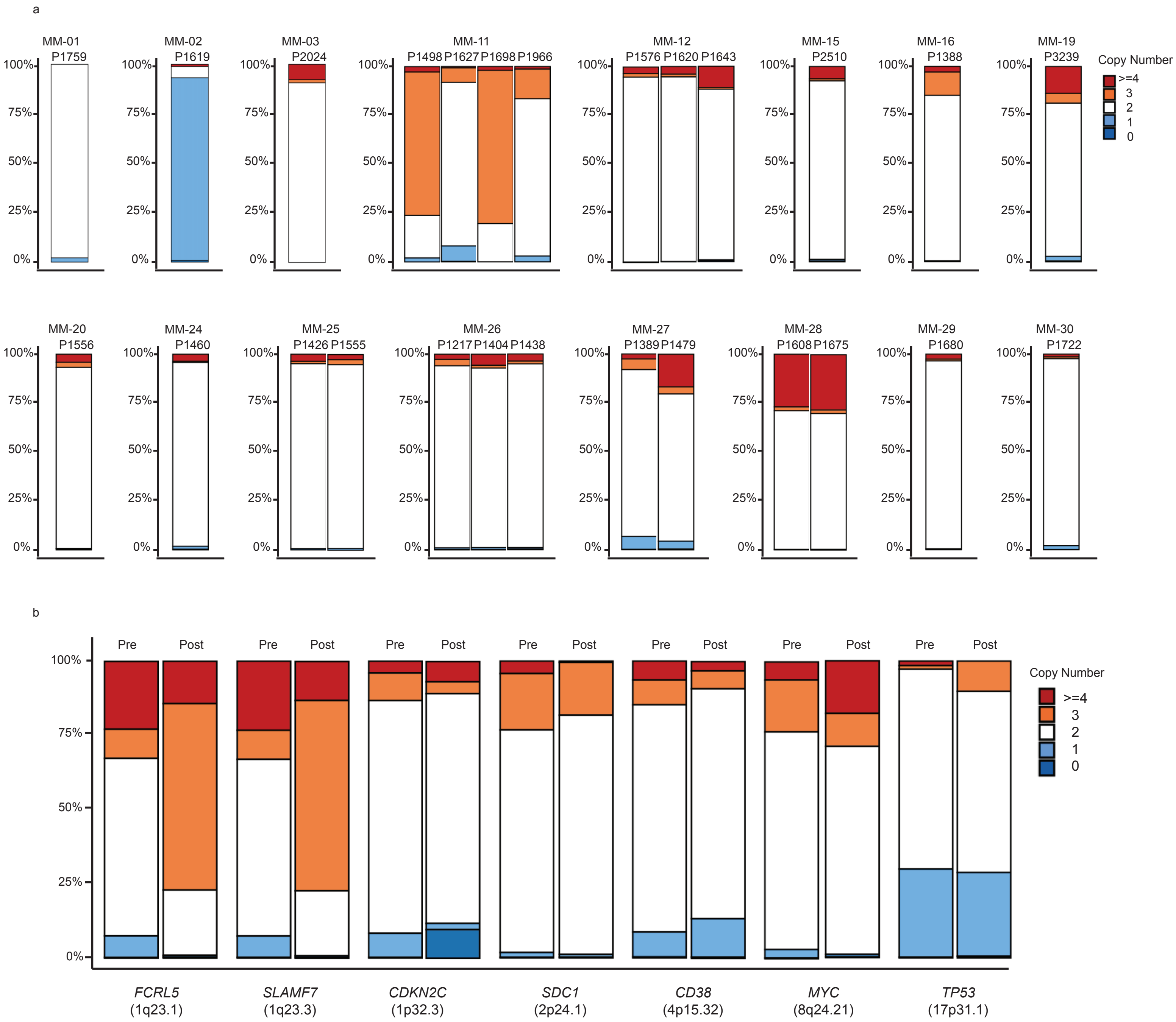

Supplementary Fig.13

A. Cell Viability of CellTrace Violet pre-stained cells (Calcein AM-FITC and Propidium Iodide-PE/ Texas Red)

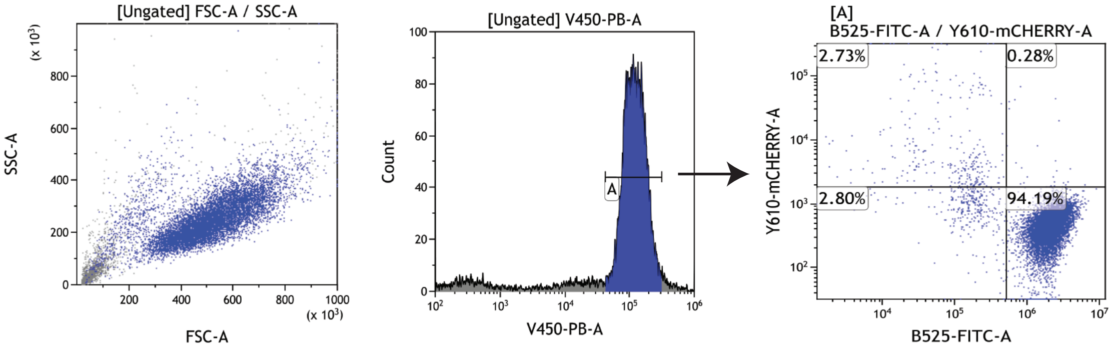

B. BCMA expression by polyclonal anti-BCMA antibody (BCMA-APC, R&D #C34557A) and monoclonal anti-BCMA antibody (BCMA-PE, Biolegend #357504)

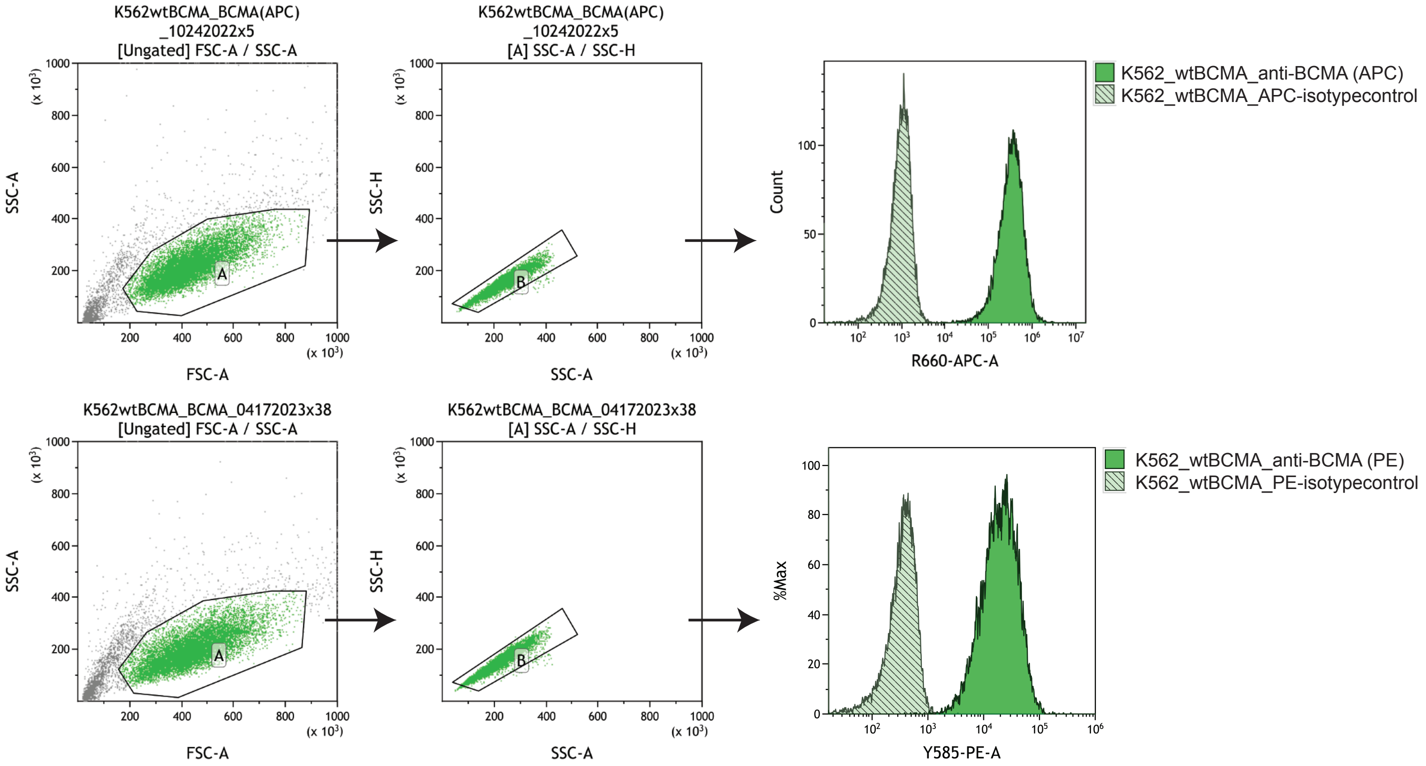

C. GPRC5D expression by anti-GPRC5D antibody (GPRC5D-PE, provided by Janssen Pharmaceutical)

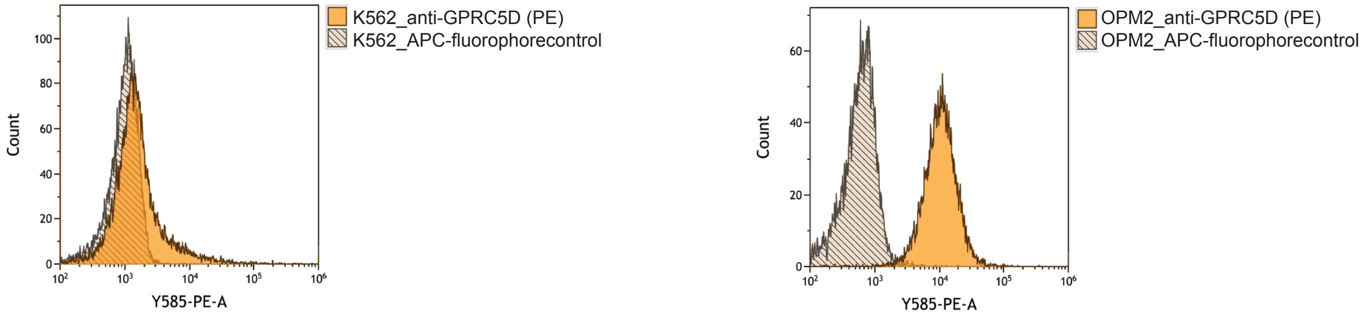

D. T cell engager Binding Assay: Anti-IgG2 antibody (IgG2-PE, Soutern Biotech #9070-09) secondary antibody staining post Elranatamab

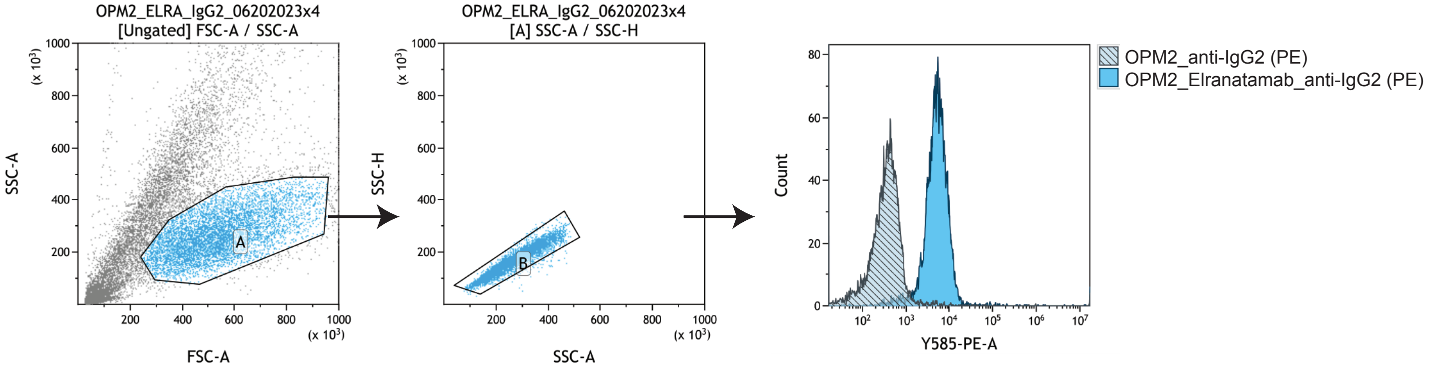

SUPPLEMENTARY FIGURE LEGENDS

Supplementary Fig. 1: scCNV analysis confirming clonality of residual BCMA positive plasma cells in case MM-01. For estimation of cancer cell fraction with copy number changes at *TNFRSF17* locus, mitotic and diploid CD138+ cells were excluded (panel a and b). (c) scCNV analysis at the *TNFRSF17* locus confirmed that 86.8% of the cells harbor biallelic loss of *TNFRSF17,* while subclones (labelled clusters 1-7) retain two copies of *TNFRSF17.* (d) Each of these clusters demonstrate clonal copy number changes in the other chromosomes, confirming their clonality.

Supplementary Fig.2: Digital polymerase chain reaction (PCR) for detection of *TNFRSF17* mutations. Digital PCR scatter plots for (a) p.Arg27Pro, (b) p.Ser30del, and (c) p.Pro34del *TNFRSF17* mutations in pre- and post-relapse CD138+ MM samples from case MM-03 and MM-17. Wild-type allele amplifications are represented by red dots while mutant allele amplifications are represented by blue dots. Green dots represent mixed wild type and mutant allele signal. Yellow dots represent wells in which no amplification occurred. (d) Limit of detection assay demonstrating target (mutant)/ total (mutant + wild type). Experiment was conducted using K562_wtBCMA and K562_R27P cell dilutions as indicated in source data. X axis corresponds to cell dilutions. Biologically independent samples (n=3). Data are presented as mean values +/- standard deviation.

Supplementary Fig.3: IGV screenshot demonstrating p.Pro33Ser mutation in *TNFRSF17* detected by WGS in case MM-04.

Supplementary Fig.4: Case MM-33 IGV screenshot demonstrating multiple independent deletions of *TNFRSF17* locus coupled with subclonal deletion of serine 30 post anti-BCMA TCE. WGS of sorted CD138+ cells (59% purity) from MM-33 post-anti-BCMA TCE identified multiple events altering *TNFRSF17*. (a) IGV screenshot sorted by base demonstrating 3 base pair deletion (GRCh38; chr16:g.11965408_11965410del) resulting in p.Ser30del in *TNFRSF17*. This in-frame deletion removes the last nucleotide of the C28 codon and first two nucleotides of the S29 codon. This in-frame deletion removes the last nucleotide of the C28 codon and first two nucleotides of the S29 codon. As a consequence of this deletion, the C28 amino acid is retained but one of the two back-to-back serine residues is deleted. (b) IGV screenshot sorted by insert size demonstrating 10 of 11 deletions overlapping the *TNFRSF17* locus. The heatmap shows the predicted copy number states with the overlapping deletions predicted to represent a mixture of clones with homozygous deletions of *TNFRSF17* in 85% of cells.

Supplementary Fig.5: Mutations in BCMA induce conformational change. (a) BCMA structural analyses demonstrated that p.Arg27Pro, p.Ser30del, and p.Pro34del induced a conformational change in the 3D structure of BCMA, particularly in the β1 strand (residues 12-15). The two deletion mutations had the highest impact on the structure of

BCMA. (b and c) Prediction outcome of the p.Arg27Pro mutation on BCMA (PDB 2KN1 PDB DOI: 10.2210/pdb2KN1/pdb) using DynaMut (Nucleic Acids Res. 2018;46:W350-W355).[1,2] Prediction of interactomic interaction comparing wild type and p.Arg27Pro BCMA. Wild type and mutant residues are colored in light green and are also represented as sticks alongside with the surrounding residues. $\Delta\Delta G$ predictions and Δ vibrational entropy of BCMA p.Arg27Pro are indicated. Protein flexibility predictions further corroborated the instability and strain induced by p.Arg27Pro in BCMA. p.Arg27Pro was predicted to destabilize the BCMA molecular structure as indicated by the SIFT (Sorting Intolerant From Tolerant) score of 0, and PolyPhen (Polymorphism Phenotyping v2) score of 1 (rs200779776 SNP Ensembl).[3]

Supplementary Fig.6: Mutations in BCMA alter the binding interface between BCMA and anti-BCMA TCEs. (a) *In silico* docking simulations between BCMA and anti-BCMA TCE demonstrated predictions that were consistent with *in vitro* TCE binding and killing assays. BCMA p.Ser30del mutation reduced the number of hydrogen bonds between BCMA and Teclistamab, while this mutation did not significantly alter the hydrogen bond network in the binding interface between BCMA and Elranatamab (b) The lower binding affinity of p.Pro34del and p.Arg27Pro BCMA mutants to Teclistamab and Elranatamab may be attributable to their structural rigidness as predicted by molecular dynamics simulations as shown.

Supplementary Fig.7: Mutations in NF-kB pathway regulators by WGS. Heatmap depicting copy number variations (CNV), single nucleotide variations (SNV), and structural variation (SV) in selected genes involved in the NFkB pathway in CD138+ MM cell samples sorted from 30 patients receiving anti-BCMA therapies. Legend as indicated in the figure.

Supplementary Fig.8: Alterations in *GPRC5D* at MM relapse post anti- GPRC5D TCE in case MM-32. (a) Circos plot of post-Talquetamab relapse CD138+ MM based on WGS. Outer track runs clockwise from chromosome 1 to Y. Inner track shows CNVs (gains in light blue, losses in salmon). Lines inside the circle represent SVs (deletions in red, duplications in green, inversions in blue, interchromosomal translocations in black). (b) Illustration of clonal and subclonal events affecting the *GPRC5D* gene on the short arm of chromosome 12 at relapse after Talquetamab.

Supplementary Fig.9: WGS analysis of MM-02 pre-therapy sample. (a) Pre-therapy circos plot of patient MM-02 based on WGS. Outer track runs clockwise from chromosome 1 to Y. Inner track shows CNVs (gains in light blue, losses in salmon). Lines inside the circle represent SVs (deletions in red, duplications in green, inversions in blue, interchromosomal translocations in black, chromothripsis in purple). (b) Illustration of chromosome 16 with monoallelic deletion 16p and a subclonal focal deletion at *TNFRSF17* locus (translocations in black, deletions in red, duplications in green, inversions in blue).

Supplementary Fig.10: WGS analysis of chromosome 12 depicting copy number alterations and structural variations at *GPRC5D* locus in CAR T/ TCE naïve patients.

Translocations in indicated in black, deletions in red, duplications in green, and inversions in blue.

Supplementary Fig.11: Baseline rates of target gene loss in CoMMpass dataset. CoMMpass dataset 3 way plots of deletions in selected genes. *TNFRSF17* (30/895: 3.35%), *GPRC5D* (118/896: 13.17%), *FCRL5* (7/896: 0.78%), and *CD38* (50/896: 5.58%)

Supplementary Fig.12: Copy number changes per scCNV. (a) Bar plots representing pre-therapy copy number changes at *TNFRSF17* locus in each patient sample (Supplementary Table 5b). (b) Bar plots illustrating the fraction (%) of CD138+ MM cells with copy number gains or losses in selected genes from pre- versus post-CAR T/ TCE patients (Supplementary Table 6).

Supplementary Fig.13: Gating strategy for flow cytometry and antibody validation. (A) Target cell viability measurement in cytotoxicity assay: CellTrace Violet positive cells were gated on in channel V450-PB-A as shown. The selected population was then plotted on a density plot of PE/Texas Red versus FITC to determine propidium iodide positive versus calcein AM positive cells. Viability was defined as % of calcein AM positive cells. (B) BCMA expression: Live cells were selected from the forward/side scatter plot and doublets were excluded based on side scatter height/ area plot. BCMA surface protein expression levels were determined based on median fluorescent intensities (MFI) on single parameter histograms (APC for polyclonal anti-BCMA antibody, PE for monoclonal 19F2 anti-BCMA antibody). Same cells were separately stained with isotype control (APC or PE) antibodies and gated using the same strategy. The median fluorescent intensities from anti-BCMA versus control antibody stained cells were compared in a histogram as shown. (C) GPRC5D expression: Same gating strategy was used as indicated in (B). Median fluorescent intensities from anti-GPRC5D (APC) versus fluorophore control antibody stained cells were compared in K562 cells (negative control) and OPM2 cells (positive control) as shown. (D) T cell engager binding assay: Live cells were selected from the forward/side scatter plot and doublets were excluded based on side scatter height/ area plot. IgG2 binding was determined based on median fluorescent intensities on single parameter histograms (PE for anti-IgG2 antibody).

SUPPLEMENTARY TABLES

Supplementary Table 1. Summary of patient characteristics and corresponding CD138+ samples subjected to scCNV analysis and bulk WGS. Numbers starting with P, MLL, or TP indicate sample identifiers and corresponds to data files deposited to GEO. *indicates that scCNV pre samples for this case were collected prior to anti-BCMA antibody drug conjugate (ADC) exposure. ** indicates that the same scCNV samples were used as pre- and post- sample for the sequential therapies in the same patient. *** indicates that the sample was enriched with normal plasma cells. **** indicates that for this liver biopsy, two separate biopsies were collected 1 week apart. Cases MM-07, 17, 18, 19, 20 were treated with two different therapies sequentially. Highlighted in red are samples included in the pre-anti-BCMA aggregate scCNV analysis in Supplementary Table 5a. Highlighted in blue are samples included in the post-anti-BCMA (Teclistamab, Elranatamab, Ide-cel) in aggregate scCNV analysis in Supplementary Table 5a. Target = other (non anti-BCMA or GPRCD TCE or CAR T).

LLT = last line of therapy (non anti-BCMA or anti-GPRC5D TCE or CAR T). HR = high cytogenetic risk. Sample source indicates the institution where samples were collected. Also shown are the clinical trials patients receiving anti-BCMA/ GPRC5D CAR T/ TCE were enrolled in, with the respective ClinicalTrials.gov Identifier numbers. Best response was determined per International Myeloma Working Group Response Criteria.[4]

Supplementary Table 2. Summary of number of patients per treatment category in the study cohort.

Supplementary Table 3. *TNFRSF17* mutations identified in samples from CoMMpass dataset.

Supplementary Table 4. *TNFRSF17* mutations identified in samples from the Munich Leukemia Laboratory (MLL) WGS dataset of 4,995 total samples.

Supplementary Table 5. Summary of *TNFRSF17* copy number changes per scCNV analysis. (a) *TNFRSF17* copy number changes in all patients pre TCE (n= 15 patients, 26 samples) and post anti-BCMA CAR T/ TCE (n=8 patients, 8 samples) in aggregate. (b) *TNFRSF17* copy number per patient in all available pre-anti-BCMA therapy samples. (c) *TNFRSF17* copy number changes per case in patients who received anti-BCMA therapy (those with available post scCNV CD138+ MM samples). In all three tables, red indicates pre-treatment samples and blue indicates post treatment samples.

Supplementary Table 6. Copy number changes in selected genes in CD138+ MM cells from all patients pre (n=15 patients, 26 samples) and post anti-BCMA and/ or GPRC5D CAR T/ TCE (n=10 patients, 10 samples) per scCNV analysis.

Supplementary Table 7. Antibodies, reagents and cell lines used in the study. (a) Flow cytometry, western blot, and immunohistochemistry antibodies and other reagents used in this study. (b) Sources of cell lines used in this study. (c) Digital PCR probes and primer sequences. (d) Sequences of oligonucleotides used for cloning

Supplementary Table 8. Absolute p-values. (a) Absolute p-values for Fig.4f. (b) Absolute p-values for Extended Data Fig.4c.

REFERENCES

1. Rodrigues CH, Pires DE, Ascher DB. DynaMut: predicting the impact of mutations on protein conformation, flexibility and stability. *Nucleic Acids Res*. Jul 2 2018;46(W1):W350-w355. doi:10.1093/nar/gky300

2. Pellegrini M, Willen L, Perroud M, et al. Structure of the extracellular domains of human and Xenopus Fn14: implications in the evolution of TWEAK and Fn14 interactions. *Febs j*. Apr 2013;280(8):1818-29. doi:10.1111/febs.12206

3. Cunningham F, Allen JE, Allen J, et al. Ensembl 2022. *Nucleic Acids Research*. 2021;50(D1):D988-D995. doi:10.1093/nar/gkab1049

4. Kumar S, Paiva B, Anderson KC, et al. International Myeloma Working Group consensus criteria for response and minimal residual disease assessment in multiple myeloma. *The Lancet Oncology*. Aug 2016;17(8):e328-e346. doi:10.1016/s1470-2045(16)30206-6
