# Supplementary material for: Mechanisms of antigen escape from BCMA- or GPRC5D-targeted immunotherapies in multiple myeloma
Source: Nat Med. 2023 Aug 31;29(9):2295–306. doi: 10.1038/s41591-023-02491-5 (PMC10504087; doi:10.1038/s41591-023-02491-5)
Supplement: Supplementary file 2 — Reporting Summary [file 41591_2023_2491_MOESM2_ESM.pdf]

Corresponding author(s): Nizar J Bahlis

Last updated by author(s): Jun 29, 2023

Reporting Summary

Nature Portfolio wishes to improve the reproducibility of the work that we publish. This form provides structure for consistency and transparency in reporting. For further information on Nature Portfolio policies, see our Editorial Policies and the Editorial Policy Checklist.

Statistics

For all statistical analyses, confirm that the following items are present in the figure legend, table legend, main text, or Methods section.

n/a	Confirmed	
☐	☒	The exact sample size (*n*) for each experimental group/condition, given as a discrete number and unit of measurement
☐	☒	A statement on whether measurements were taken from distinct samples or whether the same sample was measured repeatedly
☐	☒	The statistical test(s) used AND whether they are one- or two-sided<br>*Only common tests should be described solely by name; describe more complex techniques in the Methods section.*
☒	☐	A description of all covariates tested
☐	☒	A description of any assumptions or corrections, such as tests of normality and adjustment for multiple comparisons
☐	☒	A full description of the statistical parameters including central tendency (e.g. means) or other basic estimates (e.g. regression coefficient) AND variation (e.g. standard deviation) or associated estimates of uncertainty (e.g. confidence intervals)
☐	☒	For null hypothesis testing, the test statistic (e.g. *F*, *t*, *r*) with confidence intervals, effect sizes, degrees of freedom and *P* value noted<br>*Give P values as exact values whenever suitable.*
☒	☐	For Bayesian analysis, information on the choice of priors and Markov chain Monte Carlo settings
☒	☐	For hierarchical and complex designs, identification of the appropriate level for tests and full reporting of outcomes
☒	☐	Estimates of effect sizes (e.g. Cohen's *d*, Pearson's *r*), indicating how they were calculated

Our web collection on statistics for biologists contains articles on many of the points above.

Software and code

Policy information about availability of computer code

Data collection

Single-cell RNA-Seq library construction, alignment, and quality control: 10X Genomics NextGEM Gel Bead emulsions (Version 2.0). I
Single-cell DNA library generation for CNV single-cell suspensions: 10X Genomics Reagent Kits User Guide (CG000153).
Whole genome sequencing: detailed in Methods section

For all the single cell methods, quality control and quantification was performed using a KAPA Library Quantification qPCR kit (KAPA Biosystems) on a BioRad qPCR instrument prior to preparing a single pool containing equi-molar amounts of each library. Pool was then subjected to on-board cluster formation and sequencing on an Illumina NextSeq 500 sequencer with a high-output v2.5 150 sequencing kit for RNA-seq and 300 cycle sequencing kit for CNV-seq as per the standard Illumina protocols. After sequencing, bcl data was converted to fastq data files using the Illumina BCL2FASTQ utility. Genomic sequence reads were treated with the CellRanger suite (cellranger v3.1.0. and cellranger-dna v1.1.0 for scRNA-seq and scCNV-seq respectively) against the human reference genome GRCh38 with default parameters.
Whole genome sequencing

Full data collection is described in the Methods section.

Data analysis

Single-cell RNA-Seq library construction, alignment, and quality control: Sequencing reads were aligned using CellRanger 3.1.0 pipeline to the standard pre-built GRCh38 reference genome. Single-cell RNA-Seq computational analyses and workflows: R package Seurat v.4.0 for normalization, scaling, integration, multi-modal reference mapping, clustering, dimensionality reduction, differential expression analysis, and visualization.
Single cell copy number variation data analysis, HDF5 matrices (cnv_data.h5) and copy number files (node_unmerged_cnv_calls.bed) generated by the cellranger-dna suite v1.1.0 as well as heatmap copy number data generated Loupe scDNA (v1.1.0), were processed with custom R scripts (available in Github repository https://github.com/nbahlis/Myeloma_Immunotherapy_Antigen_Escape). For readability

purposes and copy number estimates greater than 4 were reduced to 4 and marked "≥4".

Whole genome analytical tools are detailed in Methods section. The following tools were utilized for whole genome analysis:
1) Isaac aligner (v03.16.02.19) through BaseSpace WGS app v5 (Illumina) with default parameters.
2) Single nucleotide variants (SNV) were called with Strelka Somatic Variant Caller (v2.4.7), Mutect2 (v4.1), Lancet (v1.1.0)
3) Indels were called with Mutect2 (v4.1), Manta (v0.28.0), Strelka2 (v2.9.10), Lancet (v1.1.0) and SvABA (v1.2.0).
4) Structural variants (SV) were called with Manta (v0.28.0), SvABA (v1.2.0), Lumpy (v0.3.1)
4) Copy number variations (CNV) were called using GATK4 (v4.0.8.1, Broad institute) and ASCAT (v3.1.2).
5) gnomAD database (v2.1.1)

For statistical data analysis and plotting the following tools used:
1) GraphPad Prism (v9)
2) R version 4.2.2 (2022-10-31) -- "Innocent and Trusting"

For manuscripts utilizing custom algorithms or software that are central to the research but not yet described in published literature, software must be made available to editors and reviewers. We strongly encourage code deposition in a community repository (e.g. GitHub). See the Nature Portfolio guidelines for submitting code & software for further information.

Data

Policy information about availability of data

All manuscripts must include a data availability statement. This statement should provide the following information, where applicable:

- Accession codes, unique identifiers, or web links for publicly available datasets
- A description of any restrictions on data availability
- For clinical datasets or third party data, please ensure that the statement adheres to our policy

Single cell RNA-Seq and single cell CNS datasets are available at NCBI GEO at the following accession: GSE226336 (https://www.ncbi.nlm.nih.gov/geo/query/acc.cgi?acc=GSE226336)

CCDS sequence data available from NCBI CCDS database (CCDS ID: CCDS10552.1)

CoMMpass dataset (https://research.themmrf.org): DNA and RNA sequencing data are available from dbGAP, phs000748 and the Genomic Data Commoms.

Research involving human participants, their data, or biological material

Policy information about studies with human participants or human data. See also policy information about sex, gender (identity/presentation), and sexual orientation and race, ethnicity and racism.

Reporting on sex and gender

This study consenting form did not require or include gender information. Therefore, gender information was not collected. Participant sex was determined based on their biological attribute and patient self reported sex. Sex was not considered in the study design of the clinical trials the patients were enrolled to, nor in the biological studies conducted on patients progressing on the therapies they received. Sex of the patients included in this analysis are listed in Supplementary Table 1. As per Nature policy, we did not conduct post hoc sex- and gender-based analysis. Furthermore, we did not have the appropriate patient consent to conduct such analysis.

Reporting on race, ethnicity, or other socially relevant groupings

This study consenting form did not require or include race, ethnicity or other socially relevant groupings. These patients demographics were not relevant to the analyses conducted and therefore such data was not collected.

Population characteristics

Patients enrolled in this analysis were multiple myeloma patients with relapsed and/or refractory disease. Relevant covariates for this study are summarized in supplementary table 1 and included the following:
1) Therapy (CAR vs TCE vs -),
2) Target (BCMA, GPRC5D, FcRL5, other),
3) Response to therapy they were receiving as per the International Myeloma Working group criteria (sCR, CR, VGPR, PR and MR, SD and PD),
4) Progression free survival,
5) Overall survival.

Recruitment

At University of Calgary, all patients included in this analysis were consented to donate bone marrow and peripheral blood samples after discussion and review of the consent form by their treating physician as a part of ongoing study to interrogate the genome of myeloma cells and surrounding immune cells. This particular analysis was limited for patients with relapsed refractory multiple myeloma treated with anti-BCMA CAR T or TCE, anti-GPRC5D TCE, or anti-FcRL5 TCE, as well as a small control cohort of patients receiving other salvage anti-myeloma standard therapies. Patients with bone marrow samples available prior to therapy initiation and at the time of disease progression were selected for this analysis.
For University Hospital Center Wurzburg, samples were collected from patients who rapidly achieved a deep clinical response and subsequently rapidly progressed.
The sample from city of Hope was selected based on progressive disease post TCE as part of a local personalized medicine study.

Ethics oversight

This study was approved by the Conjoint Health Research Ethics Board (CHREB) at the University of Calgary (Ethics ID: HREBA.CC-21-0248), and is consistent with and the Declaration of Helsinki.
For WGS samples collected and analyzed at University Hospital of Würzburg, review and approval was provided by the health research ethics board: Würzburg EK 8/21
For samples collected and analyzed at TGen institute, review and approval was provided by the health research ethics board, Project: Genome Wide Cancer Sequencing, WIRB (Western IRB) Protocol Number: 20160566

Note that full information on the approval of the study protocol must also be provided in the manuscript.

Field-specific reporting

Please select the one below that is the best fit for your research. If you are not sure, read the appropriate sections before making your selection.

☒ Life sciences ☐ Behavioural & social sciences ☐ Ecological, evolutionary & environmental sciences

For a reference copy of the document with all sections, see nature.com/documents/nr-reporting-summary-flat.pdf

Life sciences study design

All studies must disclose on these points even when the disclosure is negative.

Sample size

40 relapsed refractory MM patients (RRMM) treated with anti-BCMA CAR T and/ or anti-BCMAxCD3ε and/ or anti-GPRC5DxCD3ε TCE, or other novel anti-MM salvage therapies (venetoclax or CC-92480).
Twenty four patients treated with any anti-BCMA therapy (CAR T n=5, TCE n=16, both n=3) were included in this analysis.
Details of the samples collected from these patients are provided in supplemental tables 1 & 2 and extended data figure 1.
Sample size determination was not required for this analysis and manuscript as we are describing the biological and functional impact of mutations detected in target genes of CAR T and T cell engagers.

Data exclusions

no sample were excluded in the data presented.

Replication

Biological studies with K562 parental or stably transduced cells and MM cell lines were conducted in independent biological triplicates or more.
Genomic studies and flow cytometry studies with primary CD138 sorted bone marrow plasma cells were singleton experiments due to limitation of the availability of these primary cells.

Randomization

randomization was not applicable since samples were collected a posteriori from patients receiving CAR T cell or T cell engagers as part of their standard of care or as per the clinical trial they were enrolled to.

Blinding

This manuscript reports on mutations of genes targeted by myeloma immunotherapeutics, their biological and functional implications.
Genomic libraries preparation, sequencing studies and analysis were performed by personnel who were blinded to the patient disease outcome and status.

Reporting for specific materials, systems and methods

We require information from authors about some types of materials, experimental systems and methods used in many studies. Here, indicate whether each material, system or method listed is relevant to your study. If you are not sure if a list item applies to your research, read the appropriate section before selecting a response.

Materials & experimental systems

n/a	Involved in the study
☐	☒ Antibodies
☐	☒ Eukaryotic cell lines
☒	☐ Palaeontology and archaeology
☒	☐ Animals and other organisms
☒	☐ Clinical data
☒	☐ Dual use research of concern
☒	☐ Plants

Methods

n/a	Involved in the study
☒	☐ ChIP-seq
☐	☒ Flow cytometry
☒	☐ MRI-based neuroimaging

Antibodies

Antibodies used

1) PE anti-human CD269. Source:Biolegend, clone:19F2, catalog number: 357504, Lot number: B345206
2) PE mouse IgG2a,k isotype control. Source: Biolegend, clone:MOPC-173, catalog number: 400214, Lot number: B367624
3) APC anti-BCMA/TNFRSF17. Source: R&D, clone: Polyclonal, catalog number: FAB193A, Lot number:ABKL0319071
4) APC goat IgG. Source: R&D, clone: Polyclonal, catalog number: IC108A, Lot number: AAOE0621071
5) PE human BCMA/ TNFRSF17 protein, His Tag. Source: ACROBiosystems, catalog number: BCA-HP2H7
6) AF-488 mouse anti-human IgG4 Fc. Source: Southern Biotech, clone: HP6025, catalog number: 9200-30, Lot number: G1322-

VM22
7) PE mouse anti-human IgG2 Fc. Source: Southern biotech clone: HP6025, catalog number: 9070-09, Lot number: I1121-ZE31Y
8) APC anti-human IgG Fc recombinant antibody. Source: Biolegend, clone: QA19A42, catalog number: 366906, Lot number: B367022
9) Human APRIL, Fc Tag. Source: ACROBiosystems, catalog number: APL-H5268
10) PE anti-GPRC5D: Source: Janssen Pharmaceutical
11) phospho-pp44/42 MAPK (Erk 1/2) (Thr202/ Tyr204). Source: Cell Signaling, clone: polyclonal, catalog number: 4370S, Lot number: 28
12) p44/42 MAPK (Erk 1/2). Source: Cell Signaling, clone: 137F5, catalog number: 4695S, Lot number: 35
13) anti-rabbit IgG Horseradish Peroxidase. Source: Cell Signaling, clone: polyclonal, catalog number: 7074S, Lot number 30
14) anti-mouse IgG Horseradish Peroxidase. Source: Cell Signaling, clone: polyclonal, catalog number: 7076S, Lot number 29
15) anti-CD138 . Source: Agilent, clone: MI15, catalog number: GA642, Lot number 41529168
16) anti-GPRC5D. Source: Abcam, clone 6D9, catalog number: 55044, Lot number 1017750-4

Validation

Flow cytometry:
1) PE anti-human CD269 and APC anti-BCMA/TNFRSF17 flow cytometry antibodies were validated in K562 myeloid cell line (negative control) and K562 cell lines transduced with wild type BCMA (positive control). PE mouse IgG2 and APC goat IgG were used as respective isotype controls for the two antibodies.
2) PE anti-GPRC5D flow cytometry antibody was a gift from Janssen Pharmaceutical. It was validated using K562 myeloid cell line (negative control) and OPM2 myeloma cell line (positive controls).
Please refer to Supplementary Data Fig.13 for antibody validation and gating strategy

Western blot:
The validation of primary antibodies phosphor-ERK, ERK, V5, and GAPDH have been validated for western blot applications and validation information can be found on the manufacturer's website.

1) rabbit anti-phospho-pp44/42 MAPK (Erk 1/2) (Thr202/ Tyr204), Cell Signaling #4370S, 1:2000, Validated by manufacturer: https://www.cellsignal.cn/products/primary-antibodies/phospho-p44-42-mapk-erk1-2-thr202-tyr204-d13-14-4e-xp-rabbit-mab/4370, and in Nature Comm. 2023 PMCID: PMC10261012
2) rabbit anti-p44/42 MAPK (Erk 1/2), Cell Signaling #4695S, 1:2000, Validated by manufacturer: https://www.cellsignal.cn/browse/?N=4294956287&Ntk=Products&Ntt=4695&site-search-type=Products, and in Nature Comm. 2023 PMCID: PMC10261012
3) rabbit anti-GAPDH: Cell Signaling #2118S, 1:4000, Validated by manufacturer: https://www.cellsignal.com/products/primary-antibodies/gapdh-14c10-rabbit-mab/2118), and in Nat Comm 2023. PMC10264388
4) mouse anti-V5: Santa Cruz Biotechnology #sc-058052, 1:4000, Validated by manufacturer: https://datasheets.scbt.com/sc-58052.pdf

Immunohistochemistry:
1) mouse anti-CD138, Agilent, Validated by manufacturer: https://www.agilent.com/cs/library/packageinsert/public/P02445EFG_03.pdf
2) anti-GPRC5D (Abcam, clone 6D9) was validated in KMS12PE myeloma cell line as negative control and OPM2 myeloma cell line as positive control (Extended Fig 9d)

Eukaryotic cell lines

Policy information about cell lines and Sex and Gender in Research

Cell line source(s)

U266 ATCC TIB-196
K562 ATCC CCL-243
HEK 293T ATCC CRL-3216
OPM2 DSMZ ACC 50
KMS12PE DSMZ ACC 606

Authentication

These cell lines were not authenticated

Mycoplasma contamination

Cell lines were not tested for mycoplasma contamination however cells are cultured in media suplemented with 0.2 % normocin

Commonly misidentified lines
(See ICLAC register)

no commonly misidentified cell lines were used

Flow Cytometry

Plots

Confirm that:

☒ The axis labels state the marker and fluorochrome used (e.g. CD4-FITC).

☒ The axis scales are clearly visible. Include numbers along axes only for bottom left plot of group (a 'group' is an analysis of identical markers).

☒ All plots are contour plots with outliers or pseudocolor plots.

☒ A numerical value for number of cells or percentage (with statistics) is provided.

Methodology

Sample preparation	Primary MM CD138+ cells are sorted from bone marrow aspirates after Ficoll gradient separation of the mononuclear cell fraction followed by CD138+ magnetic bead incubation (Miltenyi Biotec #130-051-301) and column sorting. Details regarding sample preparation for immunostaining are described in Methods section.
Instrument	All flow cytometry experiments were conducted using the Beckman CytoFLEX flow cytometer.
Software	Kaluza Analysis Software 2.1 (Beckman Coulter)
Cell population abundance	For CD138+ sorted primary cells and flow cytometry analysis 6000-10,000 live events (based on forward and side scatter plots) were recorded where possible, barring limited primary MM cell availability from biopsy samples.<br>For studies involving cell lines (K562 or U266) 4000-10000 events were recorded.
Gating strategy	Gating for BCMA surface expression was based on viable cells based on forward and side scatter plots and doublet removal (by plotting FSC height vs FCS area). BCMA surface protein expression levels were determined based on median fluorescent intensities (MFI) on single parameter histograms. The same gating strategy was applied for GPRC5D flow cytometry analysis.<br><br>Cell viability staining: Calcein AM (Thermo Fisher #C1430) and propidium iodide (PI) (BioVision #1056) were used to stain the cells prior to flow cytometry as per manufacturer protocols. 4,000-10,000 events of CTV positive cells were collected per treatment condition. After gating on CTV positive cells in the BV421 channel, the cells were displayed on two parameter dot (density) plot (PE/ Texas Red in vertical axis for PI, and FITC in horizontal axis for calcein AM) to determine the proportion of Propidium Iodide versus calcein AM positive cells. A cluster of cells staining strongly positive for calcein AM were considered viable and their gated percentage was used to compare target cell viabilities among different treatment conditions.<br><br>Further details regarding viability staining and TCE binding are detailed in Methods section.

☒ Tick this box to confirm that a figure exemplifying the gating strategy is provided in the Supplementary Information.
