# Supplementary figures and images for: Mechanisms of antigen escape from BCMA- or GPRC5D-targeted immunotherapies in multiple myeloma
Source: Nat Med. 2023 Aug 31;29(9):2295–306. doi: 10.1038/s41591-023-02491-5 (PMC10504087; doi:10.1038/s41591-023-02491-5)

ED\_Fig4e

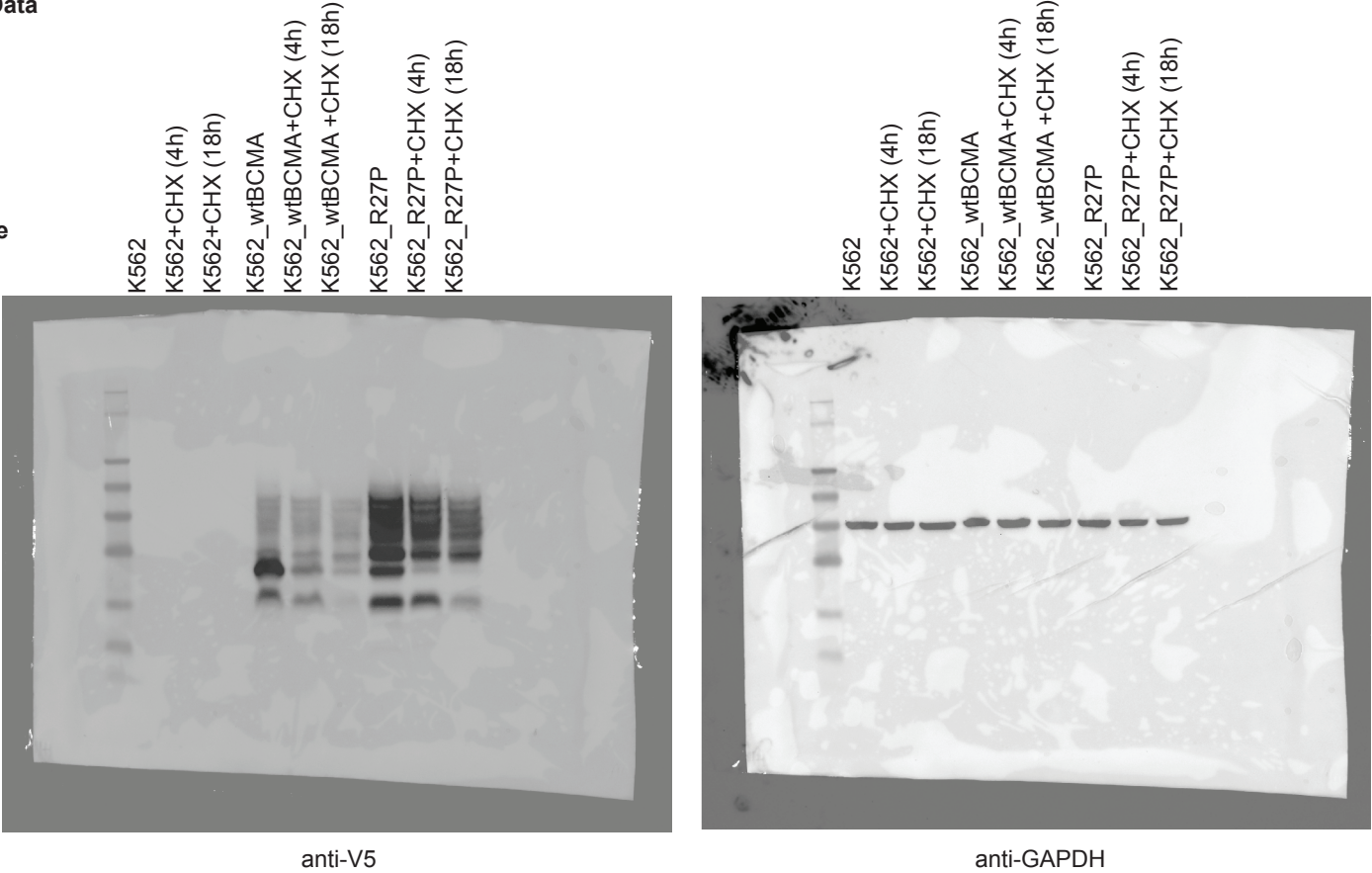

ED\_Fig7d

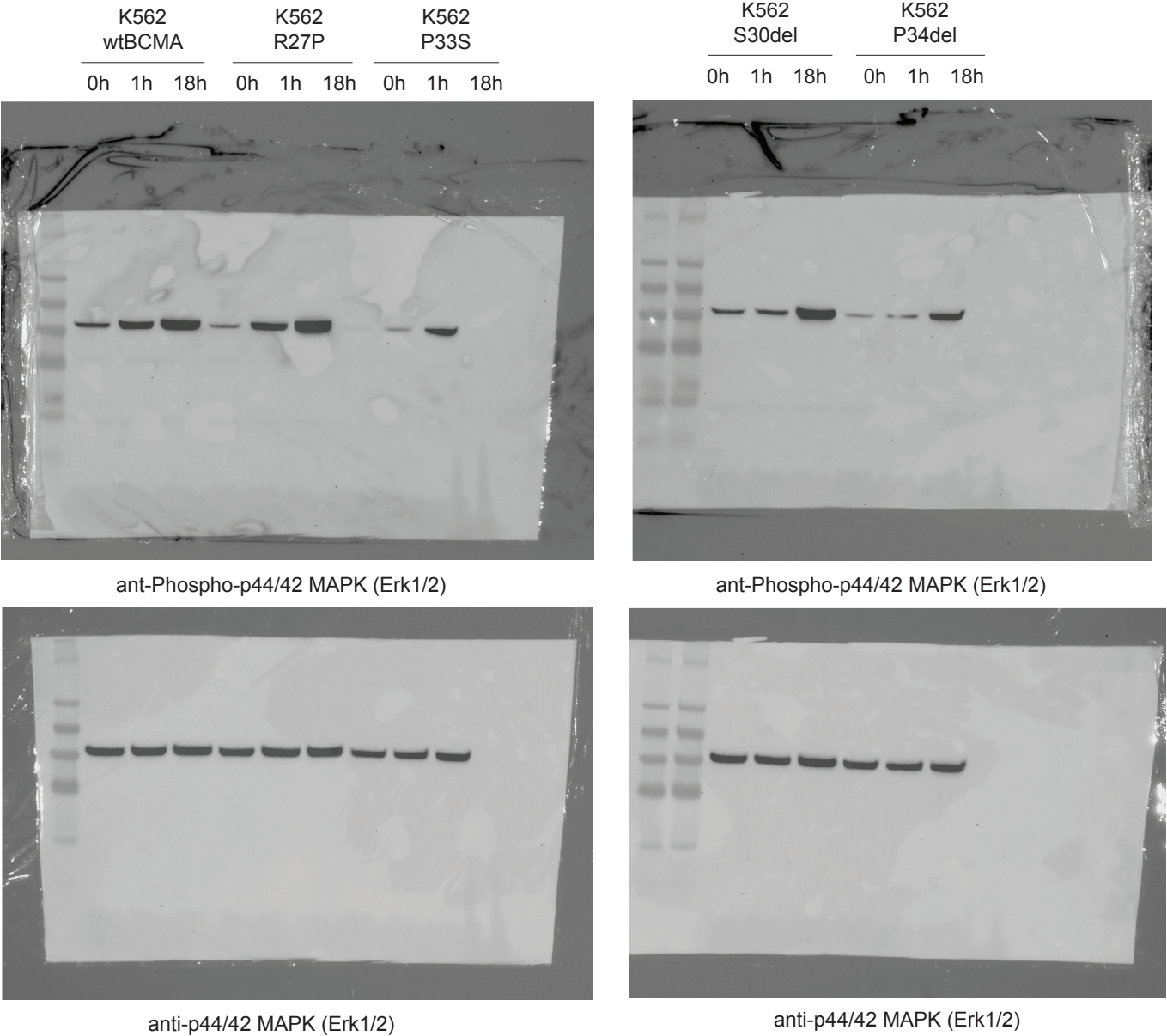

Supplement: Supplementary file 6 — Unprocessed blots for Extended Data Figs. 4e and 7d. [file 41591_2023_2491_MOESM6_ESM.pdf]
